# Molecular Tuning in Diaryl-Capped Pyrrolo[2,3-*d*:5,4-*d*′]bisthiazoles: Effects of Terminal Aryl Unit and Comparison to Dithieno[3,2-*b*:2′,3′-*d*]pyrrole Analogues

**DOI:** 10.3390/molecules27196638

**Published:** 2022-10-06

**Authors:** Eric J. Uzelac, Irene Badía-Domínguez, Spencer J. Gilman, M. Carmen Ruiz Delgado, Seth C. Rasmussen

**Affiliations:** 1 Department of Chemistry and Biochemistry, North Dakota State University, NDSU Dept. 2735, P.O. Box 6050, Fargo, ND 58108-6050, USA; 2 Department of Physical Chemistry, University of Málaga, Campus de Teatinos s/n, 29071 Málaga, Spain

**Keywords:** fused-ring heterocycles, pyrrolo[2,3-*d*:5,4-*d*′]bisthiazole, dithieno[3,2-*b*:2’,3’-*d*]pyrrole, structure–function relationships, conjugated materials

## Abstract

A series of six conjugated oligomers consisting of a central pyrrolo[2,3-*d*:5,4-*d*′]bisthiazole (PBTz) end-capped with either thienyl, furyl, or phenyl groups have been prepared from *N*-alkyl-and *N*-aryl-pyrrolo[2,3-*d*:5,4-*d*′]bisthiazoles via Stille and Negishi cross-coupling. The full oligomeric series was thoroughly investigated via photophysical and electrochemical studies, in parallel with density functional theory (DFT) calculations, in order to correlate the cumulative effects of both aryl end-groups and *N*-functionalization on the resulting optical and electronic properties. Through comparison with the analogous dithieno[3,2-*b*:2′,3′-*d*]pyrrole (DTP) materials, the effect of replacing DTP with PBTz on the material HOMO energy and visible light absorption is quantified.

## 1. Introduction

Conjugated organic materials continue to receive substantial academic and industrial interest, with a special focus on their application to technological devices such as sensors, electrochromic cells, field-effect transistors (FETs), organic photovoltaics (OPVs), and organic light-emitting diodes (OLEDs) [1,2,3,4,5]. One of the many strengths of conjugated materials is the ability to tune their electronic and optical properties at the molecular level via synthetic modification. Such molecular tuning is commonly accomplished through the choice and combinations of monomeric species applied [6], but can also involve the direct synthetic modification of specific monomeric units. This later approach can include modification of the elemental composition, the atom connectivity, or the incorporation of side chains and/or other functional groups [2,5,7,8]. In this respect, thiophene-based species have found distinct popularity due to their ease of synthetic manipulation [2,8]. 

**Figure 1:**
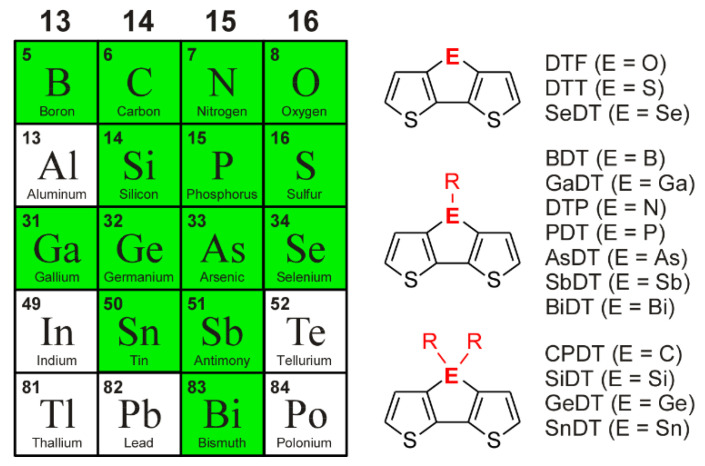
Tricyclic-fused analogues of 2,2’-bithiophene.

One successful synthetic strategy for molecular tuning has been the annulation of aromatic rings to generate various fused-ring species, with a popular approach involving the insertion of a bridging unit between the adjacent thiophene rings of 2,2′-bithiophene [9]. The most commonly applied examples of such species include cyclopenta[2,1-*b*:3,4-*b*′]dithiophene (CPDT, E = C) [8,9,10,11], silolo[3,2-*b*:4,5-*b*′]dithiophene (SiDT, E = Si) [8,9,10,11,12,13], and dithieno[3,2-*b*:2′,3′-*d*]pyrrole (DTP, E = N) [8,9,11,12,14], but a large number of such monomeric units have been generated as illustrated in Figure 1 [9]. The fused-ring nature of these units enhances the planar nature of the ground state, thus facilitating more efficient electron delocalization and a corresponding reduction in band gap [9,12,13,14]. In addition, this ring fusion reduces contributions of interannular torsional vibrations, which can decrease vibrational relaxation of the excited state and lead to increased emission [9,11]. Lastly, the bridging unit can modulate the electronics via inductive effects to tune the energies of the frontier orbitals, while the central placement of the side chains reduces potential issues of regioirregularity in the resulting polymers, while also allowing the use of relatively bulky groups without deleterious steric interactions that can result in reduced backbone planarity [11,14]. 

**Figure 2:**
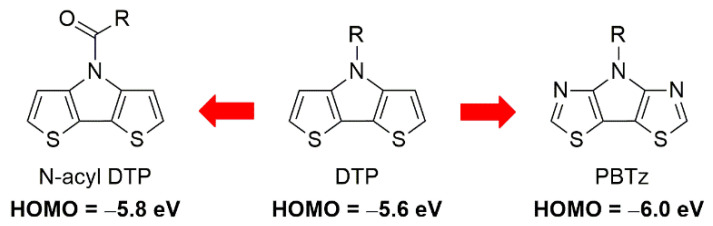
Approaches to stabilize the HOMO of DTP.

More recently, further synthetic modification of these tricyclic fused bithiophenes has included the preparation of analogues in which the flanking thiophene rings have been replaced with thiazole. Much of these efforts have been driven by a desire to stabilize the high HOMO energies typical of the species illustrated in Figure 1. This is particularly true for the highly electron-rich DTP units, where these high energy levels can limit stability and the effective application of DTP-based materials to various devices. As such, it is not surprising that the first example of these thiazole species was the DTP analogue pyrrolo[2,3-*d*:5,4-*d*′]bisthiazole (PBTz, Figure 2) [15,16,17]. Introduced by Heeney and co-workers in 2010 [15], these new PBTz units have then been applied to both small molecules [16,18] and polymeric materials [15,19,20,21,22].

**Figure 3:**
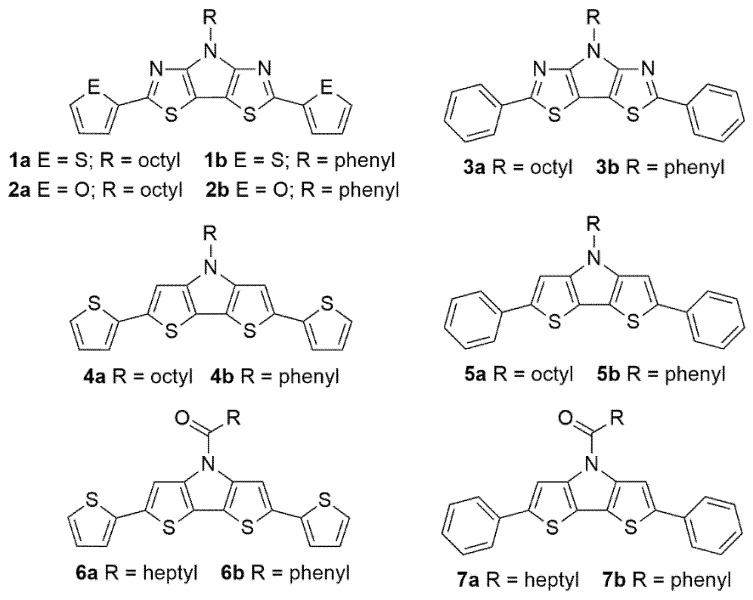
Diaryl-capped PBTz and DTP oligomers.

**Figure 4:**
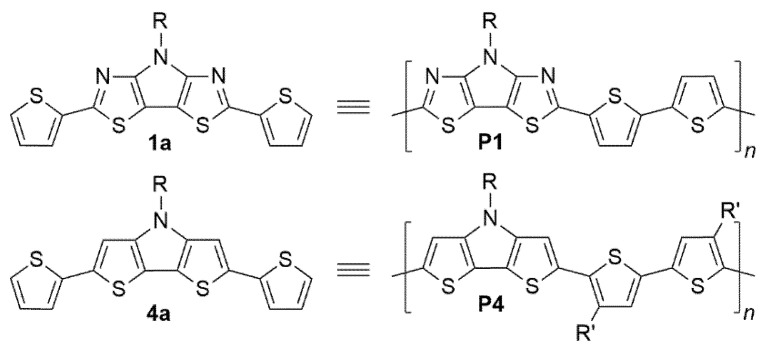
Oligomers **1a** and **4a** as models of known copolymeric materials.

As shown in Figure 2, the replacement of the thiophenes with thiazoles in PBTz successfully lowers the HOMO energy of DTP [17], resulting in even deeper HOMO energies than the alternate approach of *N*-acyl derived DTPs introduced by Evenson and Rasmussen the same year [23]. A direct comparison of PBTz to DTP, however, has also revealed that this HOMO stabilization comes at the price of a significant reduction in light absorption [17], which is critical for applications such as OPVs. To further quantify both the stabilization effect of these PBTz building blocks and the potential loss of light absorption, the diaryl-capped PBTz oligomers **1**–**3** (Figure 3) have been prepared and characterized in the current study in order to fully investigate the molecular tuning effects of both aryl endgroups and *N*-functionalities (alkyl vs. phenyl). Furthermore, oligomers **1** and **3** have been specifically chosen to allow their direct comparison to the previously reported DTP analogues **4**–**7** [24,25]. The study of such mixed oligomers can also act as suitable models for known co-polymeric materials (Figure 4) [15,26,27], which will thus provide a more accurate quantification of the critical effects of these various building blocks in realistic applications.

## 2. Results and Discussion

### 2.1. Synthesis

**Scheme 1:**
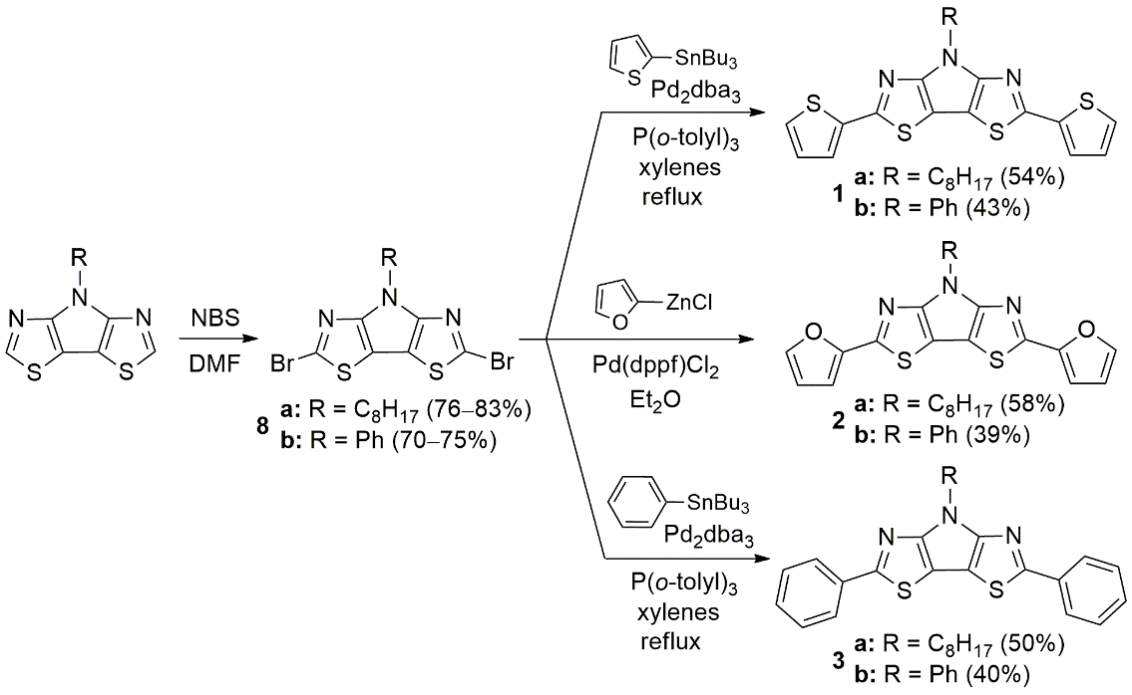
Synthesis of 2,6-diarylpyrrolo[2,3-*d*:5,4-*d*′]bisthiazoles.

Previous diaryl-capped DTPs have been synthesized primarily by Stille coupling [28], with Suzuki coupling [29] also finding application in some cases [24,25]. For general *N*-alkyl DTPs, this required generation of distannyl DTP intermediates, as the analogous dibromides are extremely reactive and are prone to readily decompose via polymerization [14,25,30]. In contrast, the deeper HOMO of the *N*-acyl DTPs allowed the generation of dibromo intermediates as stable species in high yield. As such, this approach was also applied to the PBTz monomers as outlined in Scheme 1. Bromination of either *N*-octyl and *N*-phenyl PBTz via *N*-bromosuccinimide (NBS) smoothly produces **8a** and **8b**, although in slightly lower yield than previously reported for the analogous *N*-acyl DTPs [25]. The reaction can be performed in either dimethylformamide (DMF) or chloroform, and bromination resulted in a diminishing of solution luminescence as the reaction proceeded (due to the heavy atom effect [31]), which could be used as a visual cue to monitor progress.

Synthesis of **1a** was initially attempted via Stille coupling following the methods developed by Evenson and Rasmussen for its *N*-acyl DTP analogue [25]. Thus, Stille coupling of the brominated PBTz **8a** and tributylstannylthiophene was carried out in toluene at 90 °C with a Pd(OAc)_2_/P(*o*-tolyl)_3_ precatalyst mixture. These conditions, however, produced only a small amount of the desired **1a** (ca. 5%), with the bulk of the recovered material consisting of the mono-coupled product (ca. 40%) and unreacted **8a** (20%). Even after extending the reaction time to 24 h at reflux, the ratio of the mono-coupled product to the desired **1a** was still only 2:1. Furthermore, the use of stoichiometric excesses of reactants did not make a significant difference in the ratio of products or their yields.

The limited success of the initial attempts prompted a deeper consideration of the known differences between DTP and PBTz. Whereas the final Buchwald-Hartwig amination step in the DTP synthesis could be carried out in toluene [14], attempts to synthesize PBTz under these conditions failed, after which it was found that the application of the higher boiling xylenes was necessary for the successful synthesis of PBTz [17]. As such, the initial conditions were attempted again with xylenes as a solvent, allowing a reflux temperature of ca. 137–140 °C. Under these new conditions, the reaction successfully generated **1a** as the primary product, with only a small fraction of the mono-coupled species and only a trace of unreacted **8a**. These new conditions were then successfully applied to the *N*-phenyl analogue **1b**, as well as the phenyl-capped oligomers **3a** and **3b** (Scheme 1). During the purification of these oligomers via column chromatography, it was found that residual arylstannane content had similar R*_f_* values to the desired products, making it difficult to remove them even after multiple chromatography runs. Ultimately, it was found that washing the oligomers with 10% HCl removed these troublesome impurities, after which pure materials could be successfully obtained via chromatography.

Attempts to apply Stille coupling to the synthesis of the furyl-capped PBTz oligomers **2a** and **2b** failed, however, as tributylstannylfuran decomposed under refluxing xylene. Negishi coupling was then investigated as an alternative method with the potential of more gentle, room-temperature reaction conditions [32]. Synthesis of the necessary furylzinc chloride intermediate appeared successful and the addition of **8a** to this intermediate solution resulted in the same bright fluorescence observed during the formation of the previous PBTz oligomers via Stille conditions. The furyl-endcapped PBTz oligomers **2a** and **2b** were thus produced via Negishi cross-coupling methods in reasonable yields (Scheme 1).

**Figure 5:**
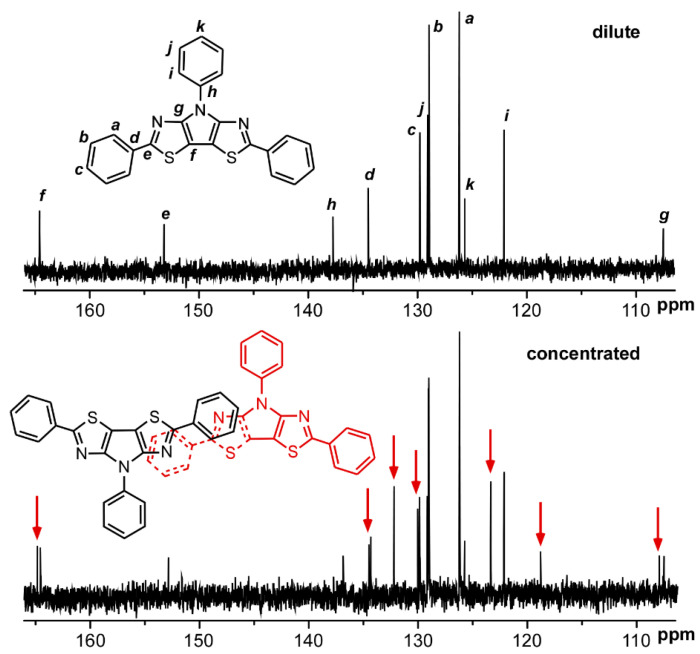
^13^C NMR spectra of oligomer **3b** in both dilute and concentrated conditions (new peaks highlighted with red arrows).

While the *N*-octyl functionalized oligomers all exhibited good solubility in organic solvents, the analogous *N*-phenyl derivatives exhibited diminished solubility. The species end-capped with either thienyl or furyl groups (**1b** and **2b**) were still soluble enough that sufficient quantities could be dissolved in CDCl_3_ for ^13^C NMR measurements at 40 ℃ without complications. The final oligomer **3b**, containing phenyl moieties at all three variable positions, however, was found to be the least soluble such that measurements at normal concentrations would not fully dissolve, even when performing measurements at the maximum scanning temperature of 50 ℃. Under such conditions, additional ^13^C resonances were found in comparison to the other analogues, which seemed to indicate potential aggregation. As shown in Figure 5, the resonances corresponding to the PBTz core and the phenyl end-caps each had a duplicate, slightly shifted in comparison to the expected values. This was ultimately explained via a proposed slipped-stack interaction between two oligomers, which would thus provide a different chemical environment for seven carbons in each molecule. Such a packing interaction had also been observed in the X-ray structure of a previous thienyl-capped DTP oligomer [24]. Repeating the measurement at low concentration (7 mg in 700 μL CDCl_3_) resulted in the loss of these additional resonances to give the expected number of carbon signals, further supporting the hypothesis of aggregated dimers at higher concentrations.

### 2.2. Electrochemistry

**Figure 6:**
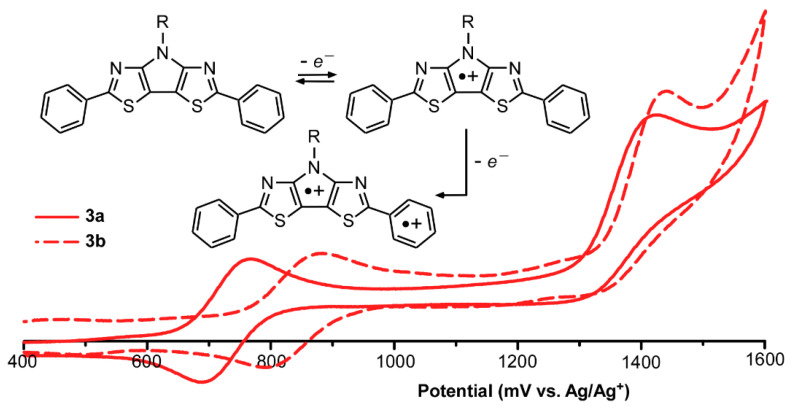
Cyclic voltammograms of the phenyl-capped PBTz oligomers **3a** and **3b**.

A primary focus of the current study is to quantify the stabilization effects of utilizing PBTz units in place of DTP in mixed oligomeric species. Thus, to evaluate the electronic effects on the frontier orbitals by replacing thiophene with thiazole, all six PBTz oligomers were characterized via cyclic voltammetry (CV). The resulting electrochemical data of the oligomers are summarized in Table 1 and representative CVs for **3a** and **3b** are given in Figure 6. 

Similar to the previously reported DTP analogues [25], all six of the PBTz oligomers exhibited an initial quasi-reversible redox couple, followed by a second irreversible oxidation at higher potential (Figure 6). Of course, the reversibility of the initial oxidation is somewhat unusual for thiophene- or furyl-capped species, as oxidation commonly results in the formation of a radical cation with significant radical density on the α-positions of the external heterocycles. Rapid coupling of the radical cations then results in larger oligomeric or polymeric species [34,35,36], thus leading to the commonly observed irreversible nature of the redox process. The reversibility of the initial oxidation in analogous DTP species has been previously attributed to the localization of the resulting radical cation to the internal fused DTP unit, with little delocalization onto the exterior capping units. This explanation was further supported by electron spin density calculations, which found that 62% of the unpaired spin was localized on the central DTP [25]. As such, the typical coupling observed for such species is only observed after the second oxidation, which introduces significant radical density on the external heterocycles. The identical electrochemical behavior observed between the DTP and PBTz oligomeric species suggests that this unusual localization is not significantly affected by the substitution of thiophene with thiazole in the central fused-ring unit. 

As with the previous DTP analogues, the choice of *N*-functionality on the central pyrrole does affect the observed potential for the first oxidation, with the initial oxidation of the *N*-phenyl derivatives (i.e., **1b**, **2b**, **3b**) observed at a higher potential than the *N*-octyl species (i.e., **1a**, **2a**, **3a**). Across the series, the shift observed from *N*-octyl to *N*-phenyl is ca. 80–110 mV, slightly greater than the ca. 60–90 mV observed for the DTP analogues [25]. In contrast, however, the effect of the *N*-functionality on the second oxidation is found to be minimal (ca. 20 mV), which further supports the proposed localization of the first and second oxidation processes. As the *N*-functionality is directly bound to the central PBTz unit, its influence on the oxidation viewed to be localized on the PBTz is more significant than that for the second oxidation, which is viewed to be remotely localized relative to the *N*-functionalization.

The choice of end-capping species also appears to affect the observed potentials of the two oxidation processes. For the first oxidation, 2-thienyl or 2-furyl endcaps result in very similar potentials, with the furyl-capped species **2a** and **2b** undergoing oxidation at slightly lower potentials (ca. 10–30 mV) in comparison to the analogous thienyl-capped species. A more significant shift is observed for the second oxidation, however, with the furyl-capped oligomers undergoing oxidation at potentials ca. 210 mV higher than the analogous thienyl-capped members. Not surprisingly, the phenyl-capped species **3a** and **3b** exhibit the highest observed potentials for both oxidation processes.

To further investigate the electronic effects, the energies of the frontier orbitals and the vertical ionization potentials (IPs) were calculated using the B3LYP, CAM-B3LYP, and PBE0 functionals, as collected in Table 2 and Table 3 (as well as Table S1 in the SI). It was found that the trends in the vertical IPs and calculated HOMO values are very similar and agree well with the trends of the experimental data. For example, larger IP values and more stabilized HOMO levels are found for the phenyl-capped oligomers, which is in good agreement with the higher potentials obtained for the first oxidation in comparison to the thienyl- or furyl-capped analogues. However, when comparing the different methods, B3LYP and PBE0 underestimate the experimentally determined HOMO energies, while CAM-B3LYP overestimate them, with PBE0 giving the best agreement with the experimental values.

**Figure 7:**
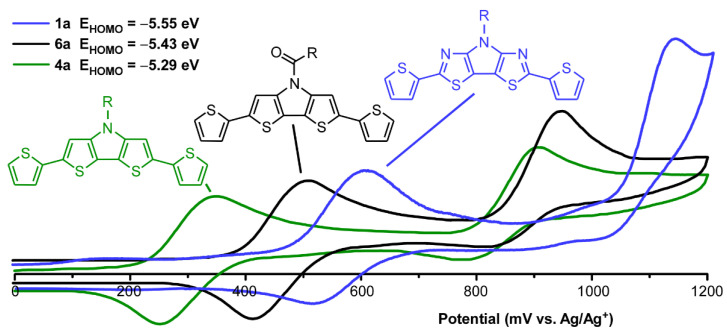
Cyclic voltammograms comparing DTP and PBTz oligomers **1a**, **4a**, and **6a**.

Of particular interest was the comparison of the PBTz species to the previous *N*-alkyl and *N*-acyl DTP analogues in order to quantify the extent of stabilization in the frontier orbitals across the series. While this had been previously determined for the monomeric species [17], it was unclear if these stabilization effects would be diluted once the fused-ring units were incorporated into larger conjugated systems. As shown in Figure 7, a clear trend can be seen in the first oxidations of the alkyl-derived series, with increasing potentials from **4a** (*N*-alkyl DTP) to **6a** (*N*-acyl DTP) to **1a** (*N*-alkyl PBTz), respectively. While this overall trend agrees with that previously found for the fused-ring monomers, the extent of stabilization of the HOMO of the oligomers is only ca. 60–70% of that found in the simple monomers. However, as the fused-ring units are technically only 50% of the total conjugated backbone, the diminished stabilizing effect could have theoretically been greater. In addition, the extent of HOMO stabilization found from **4a** to **1a** (ca. 260 meV) is in good agreement with that seen in the corresponding polymers of these oligomeric models **P4** and **P1** (ca. 320 meV, Figure 4) [15,26]. 

The trends in the second oxidation are also interesting across the series (i.e., **4a**-**6a**-**1a**). The effect of changing from *N*-alkyl to *N*-acyl is relatively small, with the second oxidation shifting ca. 40 mV. In this respect, this is in relatively good agreement with the previously observed effects of *N*-functionalization on the second oxidation. In contrast, however, the shift from *N*-acyl DTP to *N*-alkyl PBTz results in a much more dramatic shift of ca. 200 mV. Here, the electronic effect is due to the introduction of the thiazoles to the conjugated backbone, which clearly has a more direct and pronounced effect on the oxidation of the terminal end-capping units.

### 2.3. Absorption Spectroscopy

**Figure 8:**
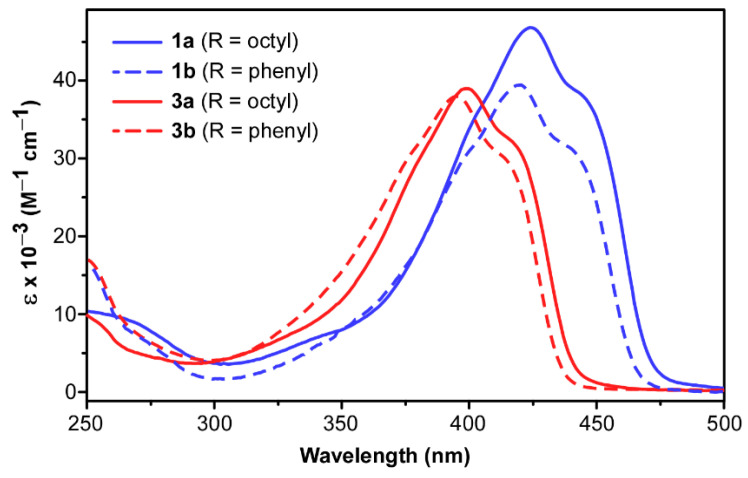
Solution UV-vis spectra of PBTz oligomers **1a**, **1b**, **3a**, and **3b**.

In addition to the stabilization effects of the PBTz unit, it is also critical to quantify any impact on the absorption properties of PBTz-based materials. As such, the full series of PBTz oligomers was analyzed via UV-vis spectrometry in both solution and thin films. The collected photophysical properties are given in Table 4 and representative solution spectra are displayed in Figure 8. 

All six PBTz species exhibit a broad π→π* transition near 400 nm, with corresponding extinction coefficients of 3.9–4.9 × 10^4^ M^−1^ cm^−1^ and oscillator strengths (*f*) of 0.72–0.91, thus corresponding to strongly allowed transitions. The absorption band also exhibits a clear vibrational structure, with shoulders observed at both higher and lower energy than the maxima. The energetic separation between the maxima and shoulders is ca. 1050–1070 cm^−1^, which is consistent with the ring-breathing mode of thiazole at 1040 cm^−1^ [37].

Across the complete series of PBTz oligomers, the *N*-phenyl derivatives (i.e., **1b**, **2b**, **3b**) exhibit a small blue-shift in absorbance in comparison to the corresponding *N*-octyl species (i.e., **1a**, **2a**, **3a**), with a corresponding decrease in absorptivity as determined by both the extinction coefficients and oscillator strengths. In this respect, the PBTz oligomers are in excellent agreement with the previous DTP analogues, which also exhibited the same trends with *N*-functionality [25]. Additional trends can also be seen based on the choice of end-capping species, with increasing absorbance red-shifts observed in the order phenyl < furyl < thienyl, with the two energetic extremes shown in Figure 8. The differences observed between the thienyl-capped and phenyl-capped species are consistent with both the previous DTP analogues and diaryl-capped 2,2′-bithiophenes [25]. The observed blue-shift for the phenyl-capped species has been attributed to various factors, with the most basic being increased steric interactions between the phenyl groups and the bithienyl core. This is somewhat supported by DFT calculations which revealed increased torsional angles between the external phenyl rings and the central DTP, with the angles of the phenyl-capped species ca. 7° greater than for the thienyl-capped analogues (see SI, Figures S18–S20). This deviation could be due to the spatial proximity of the DTP C-H at the 3- and 5-positions with the *ortho*-C-H of the phenyl groups, akin to a 1,6-diaxial interaction between the two hydrogens. However, as the C-H units of the DTP have been replaced by nitrogen in the PBTz, such an interaction would be absent in the PBTz species, yet the phenyl-capped PBTz oligomers exhibit shifts of the same magnitude as the DTP analogues. Another factor thought to play a role is the increased electron confinement in the more aromatic benzene in comparison to that of thiophene [38,39], which would reduce electronic delocalization across the molecular backbone. Unlike the steric argument, this factor would be consistent between both the DTP and PBTz oligomers and thus provides a more likely explanation.

The absorbance energies of the furyl-capped species fall in between the other two oligomer sets, although closer to the phenyl-capped species than the thienyl-capped analogues. In comparison to both thiophene and benzene, furan is the least aromatic and has the lowest electron confinement potential of the three [38,39]. As such, one could conclude that the furan-capped PBTz species would exhibit better electron delocalization and a red-shifted absorption, but this was not observed. A blue-shift in absorbance resulting from the substitution of thiophene by furan in conjugated materials, however, is consistent with various previous reports [40,41,42].

To further investigate the optical properties of the diaryl-capped PBTz series, the lowest-energy vertical excitation energies were calculated using the time-dependent DFT (TDDFT) approach. Table 5 collects the results obtained at the PBE0 level (see SI, Table S2 for those calculated at the B3LYP and CAM-B3LYP levels). The overall trends of the calculated transition energies are in good agreement with the experimental data, with the PBE0 functional providing the best accordance with the experimental data. 

**Figure 9:**
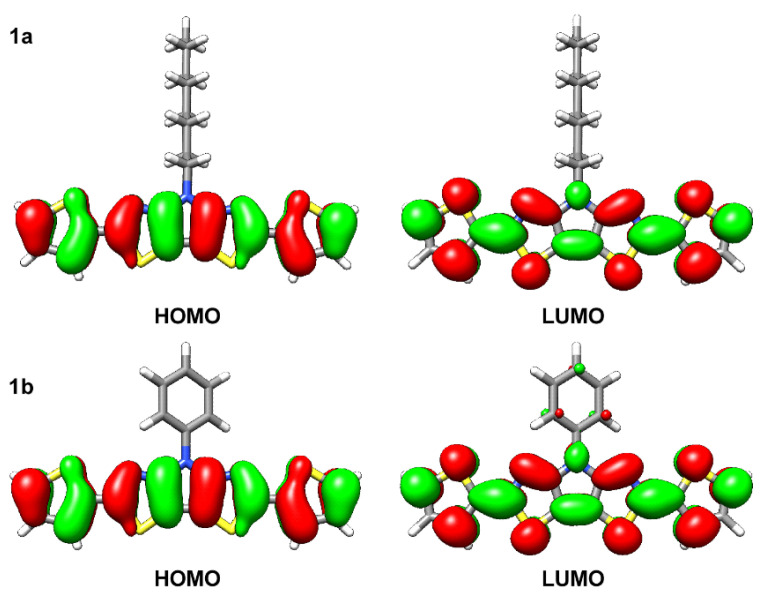
Electronic density contours, calculated at PBE0/6-31G** level, for the frontier molecular orbitals of **1a** and **1b**.

For all the PBTz oligomers, the lowest energy electronic excitation is largely attributed to a one-electron excitation from the HOMO to the LUMO. As previously seen for diaryl-capped DTP oligomers [24,25], both the HOMO and LUMO of the PBTz analogues are of π nature and spread over the entire conjugated backbone with no real contribution from the *N*-alkyl or *N*-phenyl groups (Figure 9). The observed blue-shift in absorption for the phenyl-capped oligomers is well reproduced by the theoretical calculations (i.e., the excitation energy is calculated to increase by 0.10 eV from **1a** and **3a**). In addition, theoretical calculations predict a small blue-shift upon replacing *N*-alkyl with *N*-aryl groups (i.e., 2.97 eV in **1a** and 2.98 eV **1b**), which agrees well with the experimental data and is consistent with trends previously found for the DTP analogues [25]. However, it should be pointed out that the theoretical calculations significantly overestimate the oscillator strengths in all cases.

Another primary goal of the current study was to compare the PBTz species to the previous *N*-alkyl and *N*-acyl DTP analogues in order to quantify the extent of any reduction in absorbance in PBTz-based species. As previously determined for the monomeric species, PBTz exhibits much weaker absorbance than the analogous DTPs [17]. This can be illustrated by the oscillator strength of *N*-octyl DTP (*f* = 0.50) in comparison to that of *N*-octyl PBTz (*f* = 0.29). This amounts to a ca. 42% reduction in light absorption for PBTz, which could have significant consequences in applications such as photovoltaics. Although such reduced absorbance has been reported for a number of systems when thiophene is replaced by thiazole [43,44,45,46], no significant explanation for this difference in absorptivity has been given and it was thus unclear how much this reduced absorbance would play a role once the fused-ring units were incorporated into larger conjugated systems.

**Figure 10:**
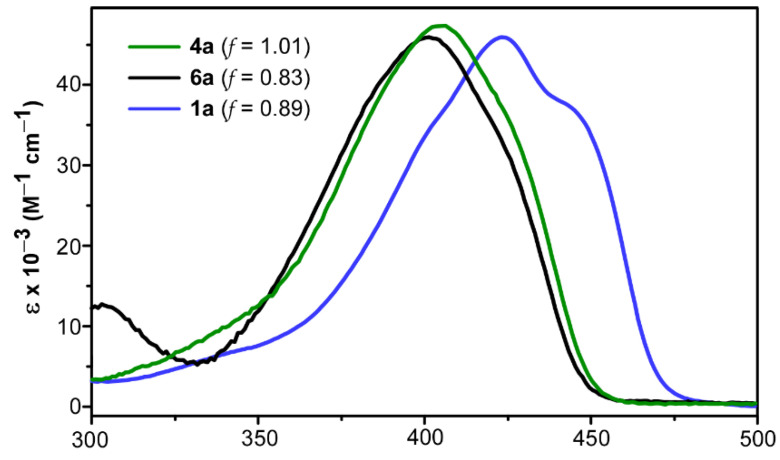
Solution UV-vis spectra of DTP species **4a** and **6a** compared to the PBTz oligomer **1a**.

To address this issue, the absorption properties of the thienyl-capped species **4a**, **6a**, and **1a** were directly compared to quantify any differences in the extent of visible light absorption. As shown in Figure 10, the PBTz oligomer **1a** exhibits a significant red-shift in absorption, with only a small decrease in extinction coefficient for its λ_max_ in comparison to the *N*-alkyl DTP analogue **4a**. Of course, the oscillator strength of the three species provides a more accurate determination of the total absorption, with the PBTz species **1a** exhibiting a value of 0.89 in comparison to 1.01 as determined for **4a**. As such, this corresponds to only a 12% reduction for the PBTz species in comparison to the DTP analogue **4a**, which is actually slightly better than the total absorbance determined for the *N*-acyl DTP species **6a** (*f* = 0.83). While the reduction in absorptivity for **6a** can be attributed to some partial charge transfer character in the electronic transition [25], no evidence of charge transfer character can be seen in the comparison of the frontier orbitals shown in Figure 9 and no absorption solvatochromism is found across a number of variable solvents (see Supporting Information). Still, it appears that the drastic reduction in absorptivity exhibited by monomeric PBTz is compensated by either pairing with other non-thiazole containing species or by simply extending the overall conjugation length.

**Figure 11:**
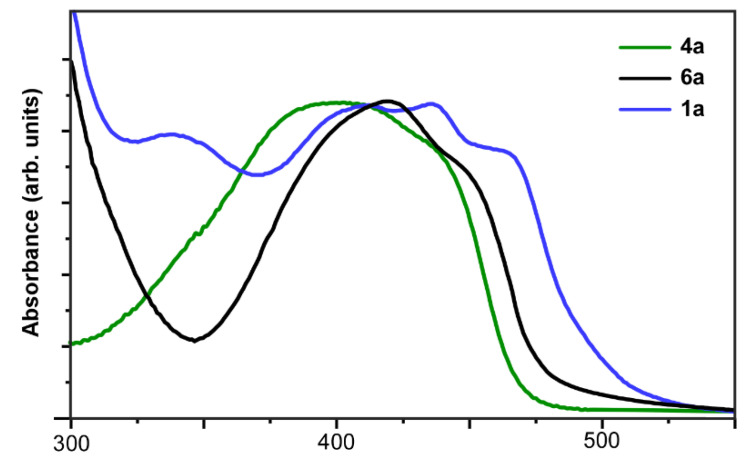
Solid state UV-vis spectra of DTP species **4a** and **6a** compared to the PBTz oligomer **1a**.

Of course, the evaluation of the absorption energies from the solution spectra given in Figure 10 is misleading as it was previously found that differences in solution confirmations can distort the real electronic trends involved. This variance in solution confirmations can be overcome by comparing the oligomers in thin films, where fully planar conformations are preferred. As shown in Figure 11, the thin film spectrum of *N*-acyl DTP species **6a** now is red-shifted in comparison to the *N*-alkyl DTP analogue **4a**, whereas the solution spectra show the opposite relationship. Still, the PBTz species **1a** is significantly red-shifted in comparison to both **4a** and **6a.** As such, the substitution of DTP by PBTz can lead to conjugated materials with better absorption in the red without the dramatic loss of light absorption that was a previous concern during the study of the simple monomers.

## 3. Materials and Methods

4-Octyl-4*H*-pyrrolo[2,3-*d*:5,4-*d*’]bisthiazole [17], 4-phenyl-4*H*-pyrrolo[2,3-*d*:5,4-*d*’]bisthiazole [17], *N*-octyl-2,6-bis(2-thienyl)dithieno[3,2-*b*:2′,3′-*d*]pyrrole (**4a**) [24], *N*-phenyl-2,6-bis(2-thienyl)dithieno[3,2-*b*:2’,3’-*d*]pyrrole (**4b**) [25], *N*-octyl-2,6-diphenyldithieno[3,2-*b*:2′,3′-*d*]pyrrole (**5a**) [25], *N*-phenyl-2,6-diphenyldithieno[3,2-*b*:2′,3′-*d*]pyrrole (**5b**) [25], *N*-octanoyl-2,6-bis(2-thienyl)dithieno[3,2-*b*:2’,3’-*d*]pyrrole (**6a**) [25], and *N*-benzoyl-2,6-bis(2-thienyl)dithieno[3,2-*b*:2’,3’-*d*]pyrrole (**6b**) [25] were prepared as previously reported. ZnCl_2_ was dried in vacuo prior to use. Dry diethyl ether and xylenes were obtained via distillation over sodium/benzophenone. Dry DMF was obtained by mixing with MgSO_4_, after which it was filtered through a silica gel plug, purged with nitrogen, and stored over molecular sieves. Dry CH_3_CN was obtained via distillation over CaH_2_. All other chemical species were reagent grade and used without further purification. All glassware for chemical synthesis was oven-dried, assembled while still hot, and cooled under dry nitrogen, with all reactions carried out under an inert atmosphere. Chromatography was performed using standard methods, with 230–400 mesh silica gel in 1-inch diameter columns. Melting points were obtained with a digital thermocouple, accurate to 0.1 °C resolution (Barnstead International, Dubuque, IA, USA). HRMS (ESI-TOF) was performed in-house (Waters, Milford, MA, USA). ^1^H and ^13^C NMR spectra were collected in CDCl_3_ on a 400 MHz spectrometer (400 MHz ^1^H, 100 MHz ^13^C) (Bruker, Billerica, MA, USA), referenced to the CHCl_3_ signal, with acquisition at 25 °C unless noted. Peak multiplicity is reported as follows: s = singlet, d = doublet, t = triplet, quint = quintet, dd = doublet of doublets, m = multiplet. See SI for copies of all NMR spectra.

### 3.1. General Procedure for Bromination of PBTz Monomers

*N*-Octyl PBTz or *N*-phenyl PBTz (1.0 mmol) was added to a 125 mL 3-neck flask and placed under a nitrogen atmosphere. Dry DMF (30 mL) was then added, followed by NBS (0.71 g, 4.0 mmol). The solution was stirred for 2 h at room temperature, during which a color change from yellow to deep red was noted. Saturated NaHCO_3_ (50 mL) was added, followed by diethyl ether (100 mL). The organic layer was separated and washed with 100 mL portions of deionized water to remove DMF. The washed organic layer was then dried over MgSO_4_, filtered, concentrated via rotary evaporation, and purified by silica gel chromatography.

#### 3.1.1. 2,6-Dibromo-4-octyl-4*H*-pyrrolo[2,3-*d*:5,4-*d*′]bisthiazole (**8a**)

The crude material was eluted with 10% chloroform in hexanes to give a white solid (76–83% yield). mp 80.8–82.1 °C. ^1^H NMR δ 4.44 (dd, *J* = 7.2 Hz, 2H), 1.95 (quint, *J* = 7.0 Hz, 2H), 1.28 (m, 10H) 0.87 (t, *J* = 6.6 Hz, 3H). ^13^C NMR δ 150.1, 132.0, 106.6, 45.7, 31.7, 30.0, 29.1, 29.0, 26.6, 22.6, 14.1. HRMS *m*/*z* 448.9203 [M^+^] (calcd for C_14_H_17_N_3_S_2_Br^79^Br^79^ 448.9231), 450.9201 [M^+^] (calcd for C_14_H_17_N_3_S_2_Br^79^Br^81^ 450.9210).

#### 3.1.2. 2,6-Dibromo-4-phenyl-4*H*-pyrrolo[2,3-*d*:5,4-*d*′]bisthiazole (**8b**)

The crude material was eluted with 10% chloroform in hexanes to give a white solid (70–75% yield). ^1^H NMR δ 8.04 (d, *J* = 7.6 Hz, 2H), 7.54 (t, *J* = 7.1 Hz, 2H), 7.35 (t, *J* = 7.1 Hz, 1H). ^13^C NMR: δ 179.2, 136.4, 132.7, 129.5, 126.8, 122.7, 108.8. HRMS *m*/*z* 415.8357 [M^+^] (calcd for C_12_H_5_N_3_S_2_Br^79^Br^81^ 415.8349).

### 3.2. General Procedure for Synthesis of Phenyl- or Thienyl-Extended PBTz Oligomers 

Thiophene or bromobenzene (5.0 mmol) was dissolved in diethyl ether (100 mL) in a 250 mL 3-neck flask. The solution was then cooled to 0 °C in an ice bath, after which BuLi (2.4 mL, 2.5 M in hexanes, 6.0 mmol) was added and the mixture was stirred for 1 h. Tributylstannyl chloride (1.63 mL, 6.0 mmol) was added, after which the solution gradually turned opaque and white. After warming to room temperature over the course of 30 min, water (50 mL) was poured into the solution, upon which the solution became clear and colorless. The organic layer was then separated, and the aqueous layer was extracted with 100 mL diethyl ether. The combined organic layers were dried over MgSO_4_, filtered into an aluminum-foil covered flask, and concentrated in vacuo without heating. The product was collected as a clear oil and used without further purification. 

Meanwhile, Pd_2_(dba)_3_ (0.010 g, 0.01 mmol), the appropriate stannyl species (1.2 mmol), tri(*o*-tolyl)phosphine (0.012 g, 0.04 mmol) and the proper brominated PBTz (0.5 mmol) were added to a Schlenk flask and placed under nitrogen. Xylenes (20 mL) were added, and the mixture refluxed overnight. A bright blue or green fluorescence gradually appeared as the reaction proceeded, indicating the formation of the product. The next day, the solution was cooled and poured into water. Diethyl ether (50 mL) was added, the organic layers separated, and the aqueous layer extracted with another 50 mL of diethyl ether. The combined organic layers were then washed twice with 50 mL aliquots of 10% HCl to remove excess arylstannane, dried over MgSO_4_, and filtered. The product was concentrated in vacuo and purified via column chromatography. 

#### 3.2.1. 2,6-Bis(2-thienyl)-4-octyl-4*H*-pyrrolo[2,3-*d*:5,4-*d*′]bisthiazole (**1a**) 

The crude material was eluted with 2% ethyl acetate/hexanes to give a yellow solid (54% yield). mp 147.4–148.2 °C. ^1^H NMR δ 7.53 (dd, *J* = 3.8, 1.1 Hz, 2H), 7.38 (dd, *J* = 5.1, 1.1 Hz, 2H), 7.09 (dd, *J* = 3.8, 5.1 Hz, 2H), 4.53 (t, *J* = 7.2 Hz, 2H) 2.06 (quint, *J* = 7.0, 2H) 1.38 (3H, m) 1.26 (m, 7H) 0.85 (t, *J* = 6.6 Hz, 3H). ^13^C NMR δ 157.4, 153.8, 138.5, 127.8, 127.0, 125.4, 104.6, 45.4, 31.8, 29.8, 29.1, 29.0, 26.6, 22.6, 13.9. HRMS *m*/*z* 457.0769 [M^+^] (calcd for C_22_H_23_N_3_S_4_ 457.077).

#### 3.2.2. 2,6-Bis(2-thienyl)-4-phenyl-4*H*-pyrrolo[2,3-*d*:5,4-*d*′]bisthiazole (**1b**)

The crude material was eluted with 20% CHCl_3_/hexanes to give a yellow solid (43% yield). mp 266.4–267.2 °C. ^1^H NMR δ 8.36 (dd, *J* = 7.8, 1.1 Hz, 2H), 7.58 (t, *J* = 7.8 Hz, 2H), 7.58 (dd, *J* = 3.6, 1.1 Hz, 2H), 7.41 (dd, *J* = 5.1, 1.1 Hz, 2H), 7.35 (dt, *J* = 7.8, 1.1 Hz, 1H), 7.10 (dd, *J* = 5.1, 3.6 Hz, 2H). ^13^C NMR δ 158.0, 152.4, 138.2, 137.4, 129.2, 128.0, 127.6, 125.9 (two overlapping signals), 122.1, 106.9. HRMS *m*/*z* 420.9819 [M^+^] (calcd for C_20_H_11_N_3_S_4_ 420.9836).

#### 3.2.3. 2,6-Diphenyl-4-octyl-4*H*-pyrrolo[2,3-*d*:5,4-*d*′]bisthiazole (**3a**)

The crude material was eluted with 20% CHCl_3_/hexanes to give a yellow solid (50% yield). mp 125.5–127.3 °C. ^1^H NMR δ 8.02 (d, *J* = 7.3 Hz, 4H), 7.44 (t, *J* = 7.3 Hz, 4H), 7.08 (t, *J* = 7.3 Hz, 2H), 4.59 (t, *J* = 7.3 Hz, 2H), 2.11 (m, 2H), 1.40 (m, 2H), 1.26 (m, 8H), 0.85 (t, *J* = 5.7 Hz, 3H). ^13^C NMR δ 164.0, 154.3, 134.7, 129.6, 128.9, 126.1, 105.1, 45.3, 31.9, 29.9, 29.2, 29.1, 26.7, 22.7, 14.1. HRMS *m*/*z* 445.1642 [M^+^] (calcd for C_26_H_27_N_3_S_2_ 445.1646). 

#### 3.2.4. 2,4,6-Triphenyl-4*H*-pyrrolo[2,3-*d*:5,4-*d*′]bisthiazole (**3b**)

The crude material was eluted with diethyl ether to give a yellow solid (40% yield). mp 269.1–270.0 °C. ^1^H NMR (Obtained at 45 °C) δ 8.46 (d, *J* = 7.8 Hz, 2H), 8.05 (d, *J* = 7.3 Hz, 4H), 7.60 (t, *J* = 7.8 Hz, 2H), 7.45 (m, 6H), 7.35 (t, J = 7.3 Hz, 1H). ^13^C NMR (Obtained at 45 °C) δ 164.6, 153.2, 137.8, 134.5, 129.8, 129.1, 128.9, 126.2, 125.7, 122.1, 107.6. HRMS *m*/*z* 410.0796 [M+H^+^] (calcd for C_24_H_16_N_3_S_2_ 410.0786).

### 3.3. General Procedure for Synthesis of Furyl-Extended PBTz Oligomers

Furan (0.07 mL, 1.0 mmol) and 30 mL diethyl ether were added to a 125 mL round bottom flask. The flask was cooled to 0 °C, BuLi (0.40 mL, 2.5 M in hexanes, 1.0 mmol) added, and the clear solution was stirred for 30 min. ZnCl_2_ (0.136 g, 1.0 mmol) was then added, the ice bath was removed, and the solution was allowed to warm to room temperature over the course of 1 h. Brominated PBTz (0.25 mmol) was added, followed by Pd(dppf)Cl_2_ (0.010 g, 5 mol%). The solution was stirred overnight, after which water (25 mL) was then added, and the organic layer separated. The aqueous layer was extracted with diethyl ether (2 × 25 mL), after which the combined organic layers were dried over MgSO_4_, concentrated in vacuo, and purified via column chromatography.

#### 3.3.1. 2,6-Bis(2-furyl)-4-octyl-4*H*-pyrrolo[2,3-*d*:5,4-*d*′]bisthiazole (**2a**)

The crude material was eluted with hexanes to give a yellow solid (58% yield). ^1^H NMR δ 7.53 (d, *J* = 1.6 Hz, 4H), 7.00 (d, *J* = 4.0 Hz, 2H), 6.57 (dd, *J* = 4.0, 1.6 Hz, 2H), 4.55 (m, 2H), 2.05, 1.36 (m, 2H), 1.24 (m, 8H), 0.85 (t, *J* = 5.7 Hz, 3H). ^13^C NMR δ 154.2, 153.6, 149.5, 143.3, 112.4, 108.7, 104.6, 45.3, 31.8, 29.9, 29.2, 29.1, 26.7, 14.1. HRMS *m*/*z* 426.1305 [M+H^+^] (calcd for C_22_H_24_N_3_S_2_O_2_ 426.1310).

#### 3.3.2. 2,6-Bis(2-furyl)-4-phenyl-4*H*-pyrrolo[2,3-*d*:5,4-*d*′]bisthiazole (**2b**)

The crude material was eluted with hexanes to give a yellow solid (39% yield). ^1^H NMR δ 8.31 (d, *J* = 8.0 Hz, 2H), 7.56 (t, *J* = 8.0 Hz, 2H), 7.54 (d, *J* = 1.6 Hz, 2H), 7.60 (t, *J* = 7.3 Hz, 2H), 7.35 (t, *J* = 8.0 Hz, 1H), 7.06 (d, *J* = 4.0 Hz, 2H), 6.56 (dd, *J* = 4.0, 1.6 Hz, 2H). ^13^C NMR δ 154.1, 153.2, 149.5, 143.4, 137.4, 129.2, 125.9, 122.4, 112.4, 109.0, 106.9. HRMS *m*/*z* 390.0323 [M+H^+^] (calcd for C_22_H_12_N_3_S_2_O_2_ 390.0321). 

### 3.4. Theoretical Methodology

The molecular geometries of all systems were optimized at the framework of the density functional theory (DFT) level using different functionals implemented in the GAUSSIAN16 program [47]. The calculations were computed using B3LYP [48,49], PBE0 [50] and CAM-B3LYP [51], in conjunction with the 6-31G** basis set [52]. On the resulting ground-state optimized geometries, harmonic frequency calculations were performed at the same level of theory to ensure finding the global minimum. The time-dependent DFT (TD-DFT) approach [53,54] was used to calculate the vertical electronic excitation energies. UCSF CHIMERA 1.11.2 software [55] was used for the visualization and analysis of molecular structures and related data such as molecular orbitals. The vertical ionization potentials (IPs) were calculated as the difference between the energy of the cation at the neutral geometry and that of the neutral species at the neutral geometry. Single-point calculations were also performed using a more flexible basis set augmented with diffuse functions (6-31++G**) [56].

### 3.5. Absorption Spectroscopy

UV-vis spectroscopy was performed on a dual beam scanning UV-vis-NIR spectrophotometer (Varian, Palo Alto, CA, USA) using samples prepared as dilute CHCl_3_ solutions in quartz cuvettes or as thin films on glass slides. The CHCl_3_ used for solution measurements was dried over molecular sieves prior to use. Oscillator strengths were determined from the visible spectra via spectral fitting to accurately quantify the area of each transition and then calculated using literature methods [57]. 

### 3.6. Electrochemistry

All electrochemical methods were performed utilizing a three-electrode cell consisting of platinum disc working electrode, a platinum wire auxiliary electrode, and a Ag/Ag^+^ reference electrode (0.251 V vs. SCE) [58]. Supporting electrolyte consisted of 0.10 M tetrabutylammonium hexafluorophosphate (TBAPF_6_) in dry CH_3_CN. Solutions were deoxygenated by sparging with argon prior to each scan and blanketed with argon during the measurements. All measurements were collected at a scan rate of 100 mV/s. Frontier orbital (E_HOMO_ and E_LUMO_) energy values were estimated from the onsets of the first oxidation or reduction in relation to ferrocene (50 mV vs. Ag/Ag^+^), using the value of 5.1 eV vs. vacuum for ferrocene [33].

## 4. Conclusions

A series of diaryl-capped PBTz oligomers have been synthesized and characterized in terms of their optical and electronic properties. Of particular interest was the direct comparison of these PBTz species with their DTP analogues in order to determine the extent of HOMO stabilization and any potential reduction in absorptivity as previously found in the comparison of the simple monomers. In terms of the HOMO stabilization, the trends found agree with that previously found for the monomers (*N*-alkyl DTP < *N*-acyl DTP < *N*-alkyl PBTz), although the extent of HOMO stabilization in the oligomers is only ca. 60–70% that found in the isolated monomers. Still, this provides stabilization of ca. 260 meV compared to *N*-alkyl DTPs and is in good agreement with the stabilization found in comparable conjugated polymers of DTP and PBTz. In terms of any differences in the extent of visible light absorption, it was found that while PBTz oligomers did exhibit decreased absorption in comparison to *N*-alkyl DTP analogues, this amounted to only a 12% reduction, considerably less than the 42% reduction found for the corresponding monomers, and comparable to the differences found in *N*-alkyl vs. *N*-acyl DTP species. Overall, when applied to conjugated materials, PBTz offers significant HOMO stabilization, red-shifted absorption, and only minimal loss in absorptivity in comparison to DTP. As such, it offers significant promise for the development of conjugated materials for technological applications such as photovoltaics.

## Figures and Tables

**Table 1:** Electrochemical data for *N*-octyl- and *N*-phenyl-2,6-diarylpyrrolo[2,3-*d*:5,4-*d*′]bisthiazoles.

Oligomer	*E*_½_^0/+1^ (V) ^1^	Δ*E* (mV) ^1^	*E*_pa_^+1/+2^ (V) ^1^	E_HOMO_ (eV) ^2^
**1a**	0.57	80	1.14	−5.55
**1b**	0.67	80	1.16	−5.65
**2a**	0.56	80	1.35	−5.54
**2b**	0.64	80	1.37	−5.62
**3a**	0.73	80	1.42	−5.71
**3b**	0.84	70	1.44	−5.82

^1^ All measurements in dry CH_3_CN containing 0.10 M TBAPF_6_. All potentials vs. Ag/Ag^+^. ^2^ E_HOMO_ = −(E_[ox vs. Fc+/Fc]_ + 5.1) (eV) [33].

**Table 2:** DFT-calculated frontier orbital energies.

Oligomer	HOMO (eV)		LUMO (eV)	
	B3LYP/6-31G**	PBE0/6-31G**	B3LYP/6-31G**	PBE0/6-31G**
**1a**	−4.96	−5.21	−1.97	−1.85
**1b**	−5.10	−5.35	−2.08	−1.99
**2a**	−4.85	−5.10	−1.75	−1.63
**2b**	−5.00	−5.25	−1.89	−1.78
**3a**	−5.07	−5.32	−1.82	−1.73
**3b**	−5.22	−5.48	−1.94	−1.85

**Table 3:** DFT-calculated vertical ionization potentials.

Oligomer	Vertical Ionization Potential (eV) ^1^	
	B3LYP/6-31G**	PBE0/6-31G**
**1a**	6.25 (5.58)	6.37 (5.84)
**1b**	6.36 (5.70)	6.49 (5.96)
**2a**	6.18 (5.43)	6.29 (5.68)
**2b**	6.30 (5.56)	6.42 (5.82)
**3a**	6.37 (5.62)	6.48 (5.89)
**3b**	6.49 (5.74)	6.62 (6.02)

^1^ The values in parenthesis correspond to the calculations at B3LYP/6-31++G**//B3LYP/6-31G* level and PBE0/6-31++G**//PBE0/6-31G* level, respectively.

**Table 4:** Photophysical data for *N*-octyl- and *N*-phenyl-2,6-diarylpyrrolo[2,3-*d*:5,4-*d*′]bisthiazoles.

Oligomer	λ_max_ (nm, CHCl_3_)	ε (M^−1^ cm^−1^)	*f*	λ_max_ (nm, film) ^1^
**1a**	423	46,600	0.89	435, (463)
**1b**	421	39,400	0.72	406, (445)
**2a**	406	48,700	0.91	395, (431), (454)
**2b**	405	40,000	0.73	426, (451)
**3a**	399	39,000	0.79	399, (420), (448)
**3b**	396	38,600	0.77	395, (413), (441)

^1^ Values in parentheses represent distinct shoulders.

**Table 5:** DFT-calculated vertical excitation energies.

Oligomer.	PBE0/6-31G**		
	E_max_ (eV)	*f*	Description
**1a**	2.97	1.28	HOMO → LUMO (97%)
**1b**	2.98	1.24	HOMO → LUMO (97%)
**2a**	3.12	1.33	HOMO → LUMO (100%)
**2b**	3.11	1.27	HOMO → LUMO (100%)
**3a**	3.17	1.34	HOMO → LUMO (100%)
**3b**	3.19	1.28	HOMO → LUMO (100%)

## Data Availability

The data presented in this study are available either within the article or the associated supplementary material.

## Supplementary Materials

The following supporting information can be downloaded at: https://www.mdpi.com/article/10.3390/molecules27196638/s1, Figures S1–S16: ^1^H NMR and ^13^C NMR spectra; Figure S17: Rotational analysis calculated at the B3LYP/6-31G** level; Figure S18: Optimized structures calculated at the B3LYP/6-31G** level; Figure S19: Optimized structures calculated at the CAM-B3LYP/6-31G** level; Figure S20: Optimized structures calculated at the PBE0/6-31G** level; Table S1: DFT-calculated frontier orbital energies and vertical ionization potentials at the CAM-B3LYP/6-31G** level; Figure S21: Frontier orbital energies calculated at the B3LYP/6-31G** level; Figure S22: Frontier orbital energies calculated at the CAM-B3LYP/6-31G** level; Figure S23: Frontier orbital energies calculated at the PBE0/6-31G** level; Figure S24: Molecular orbital topologies calculated at the B3LYP/6-31G** level; Figure S25: Molecular orbital topologies calculated at the CAM-B3LYP/6-31G** level; Figure S26: Molecular orbital topologies calculated at the PBE0/6-31G** level; Table S2: DFT-calculated vertical excitation energies at the B3LYP/6-31G** and CAM-B3LYP/6-31G** levels; Figure S27: Simulated UV-visible spectra at B3LYP/6-31G**, CAM-B3LYP/6-31G** and PBE0/6-31G** levels; Figure S28: Solution UV-visible spectra of **1a** in various solvents.
