# Supplementary material for: Molecular Tuning in Diaryl-Capped Pyrrolo[2,3-d:5,4-d′]bisthiazoles: Effects of Terminal Aryl Unit and Comparison to Dithieno[3,2-b:2′,3′-d]pyrrole Analogues
Source: Molecules. 2022 Oct 6;27(19):6638. doi: 10.3390/molecules27196638 (PMC9571600; doi:10.3390/molecules27196638)
Supplement: Supplementary file 1 [file molecules-27-06638-s001.zip › molecules-1956364-supplementary.pdf]

Molecular Tuning in Diaryl-capped Pyrrolo-[2,3-*d*:5,4-*d′*]bisthiazoles: Effects of Terminal Aryl Unit and Comparison to Dithieno[3,2-*b*:2′,3′-*d*]pyrrole Analogues

Eric J. Uzelac, Irene Badía-Domínguez, Spencer J. Gilman, M. Carmen Ruiz Delgado and Seth C. Rasmussen *

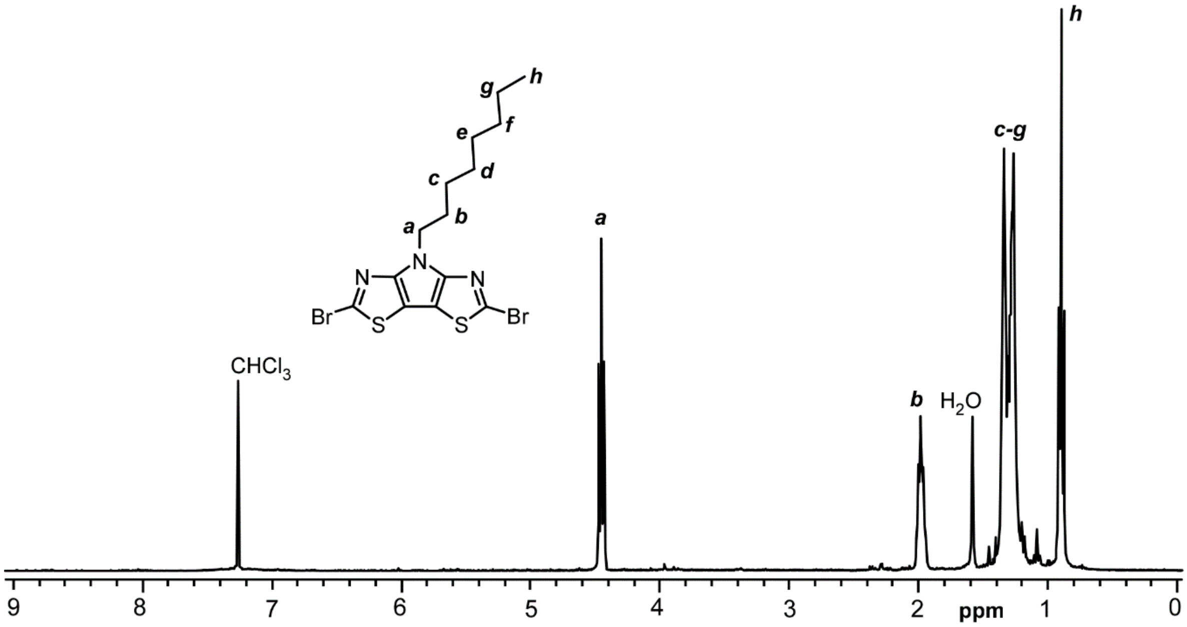

Figure S1. ^{1}H NMR Spectrum of 2,6-dibromo-4-octyl-4*H*-pyrrolo[2,3-*d*:5,4-*d′*]bisthiazole.

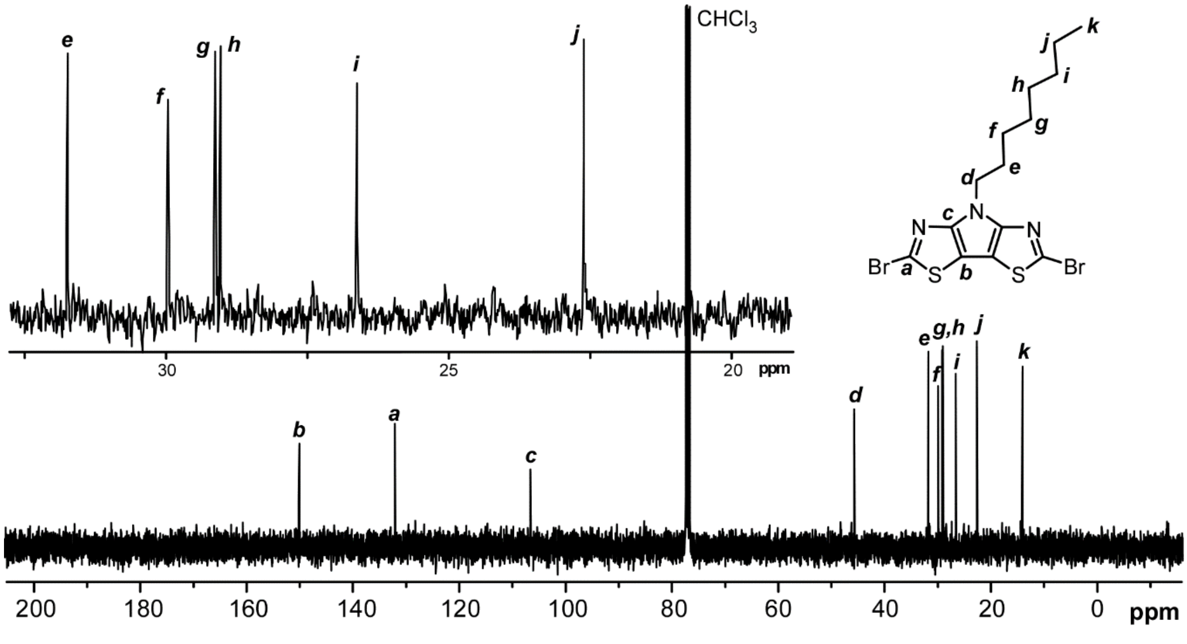

Figure S2. ^{13}C NMR Spectrum of 2,6-dibromo-4-octyl-4*H*-pyrrolo[2,3-*d*:5,4-*d′*]bisthiazole.

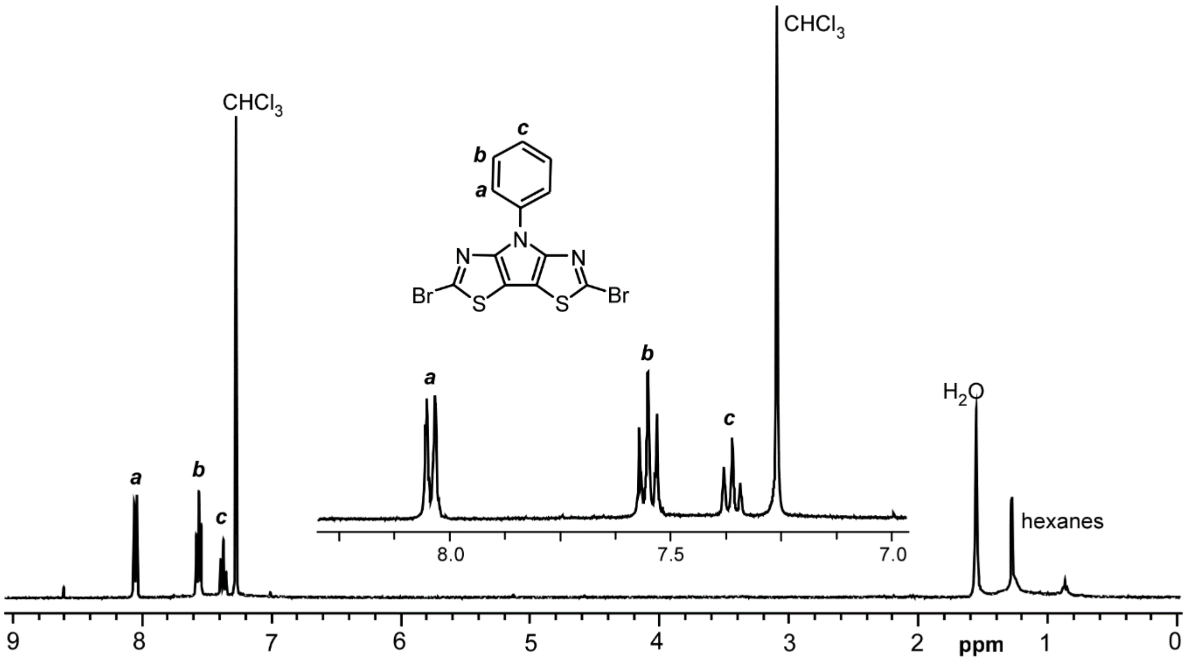

Figure S3. ^{1}H NMR Spectrum of 2,6-dibromo-4-phenyl-4*H*-pyrrolo[2,3-*d*:5,4-*d'*]bisthiazole.

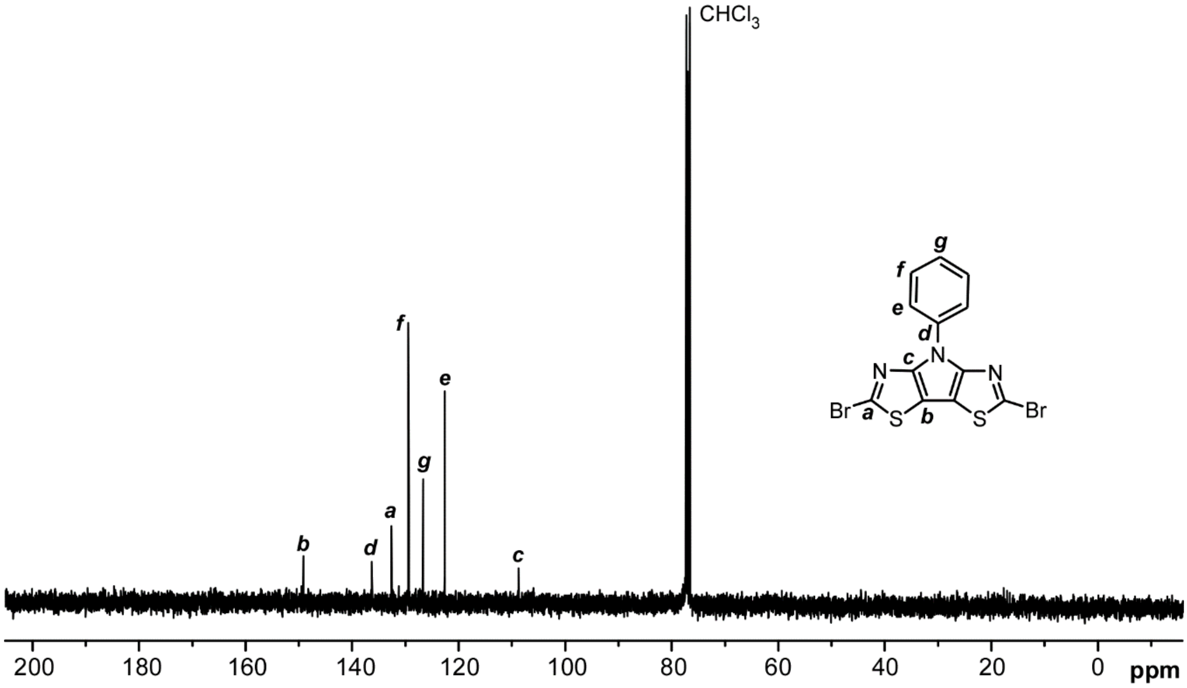

Figure S4. ^{13}C NMR Spectrum of 2,6-dibromo-4-phenyl-4*H*-pyrrolo[2,3-*d*:5,4-*d'*]bisthiazole.

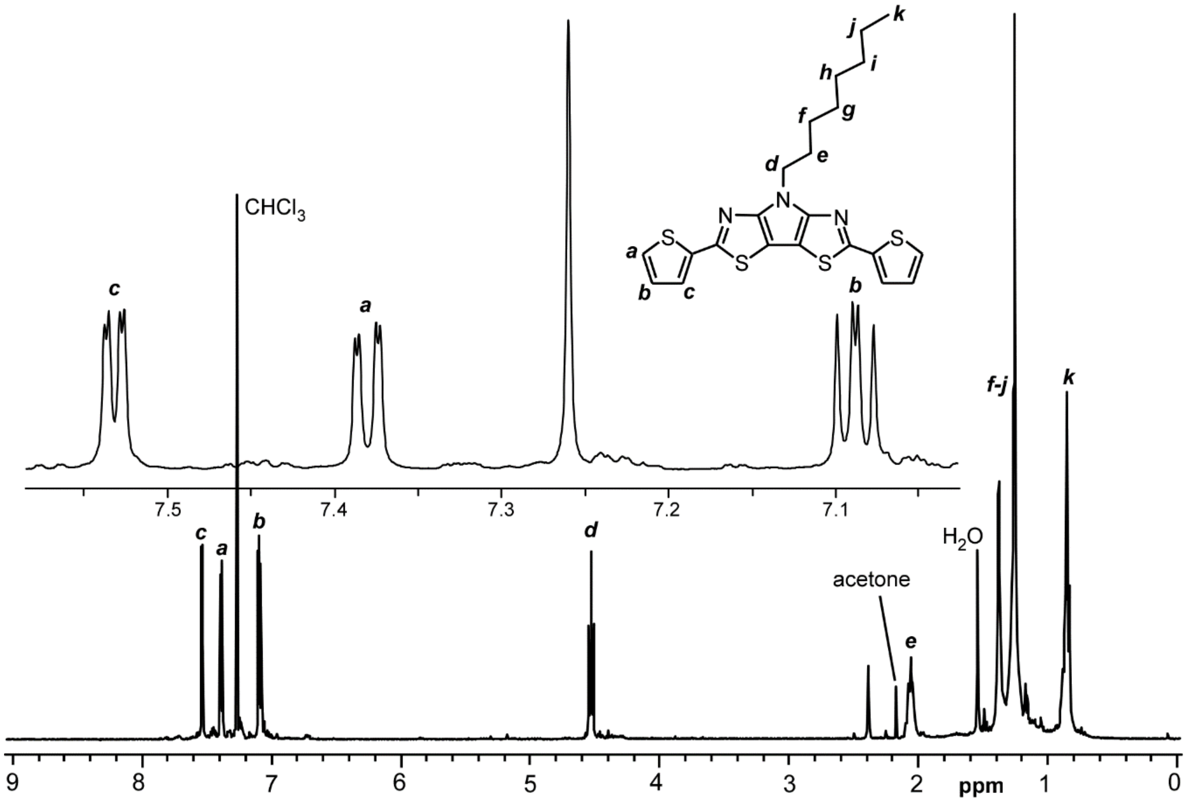

Figure S5. ^{1}H NMR Spectrum of 4-octyl-2,6-bis(2-thienyl)-4*H*-pyrrolo[2,3-*d*:5,4-*d'*]bisthiazole.

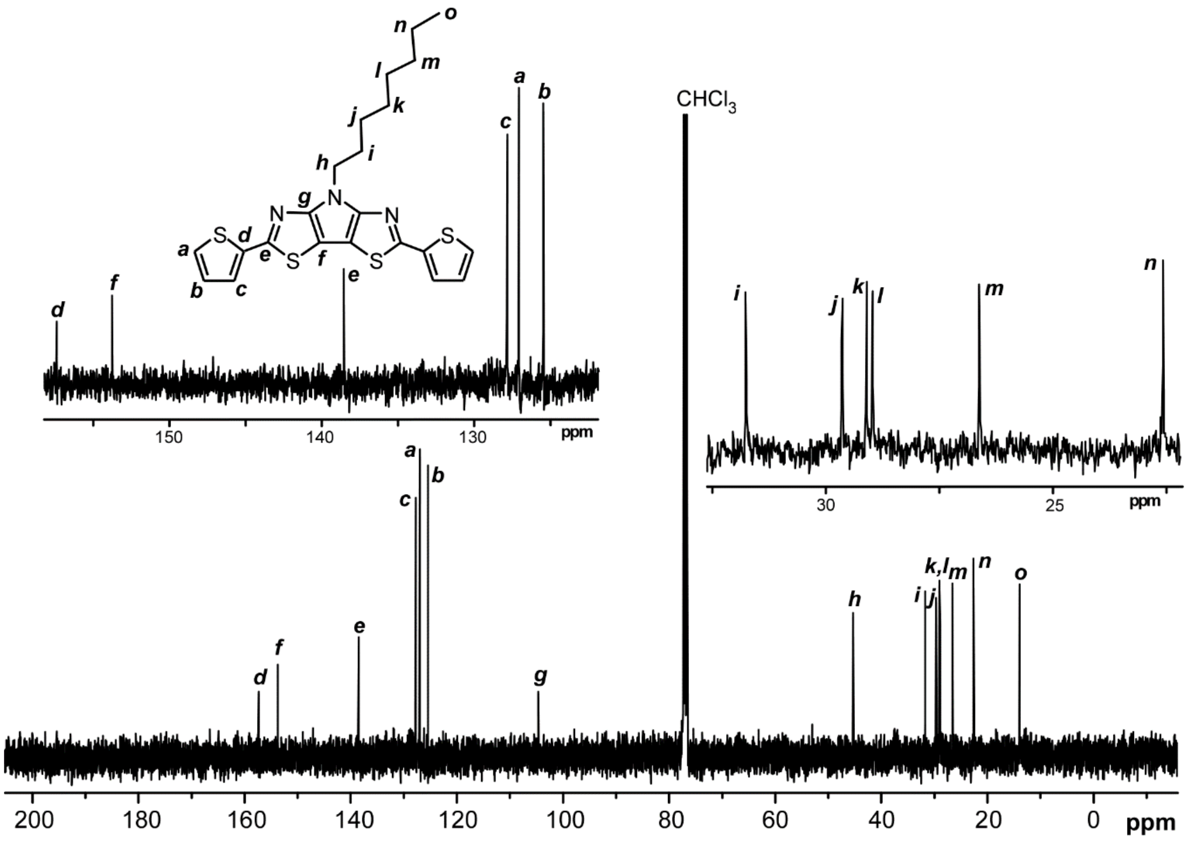

Figure S6. ^{13}C NMR Spectrum of 4-octyl-2,6-bis(2-thienyl)-4*H*-pyrrolo[2,3-*d*:5,4-*d'*]bisthiazole.

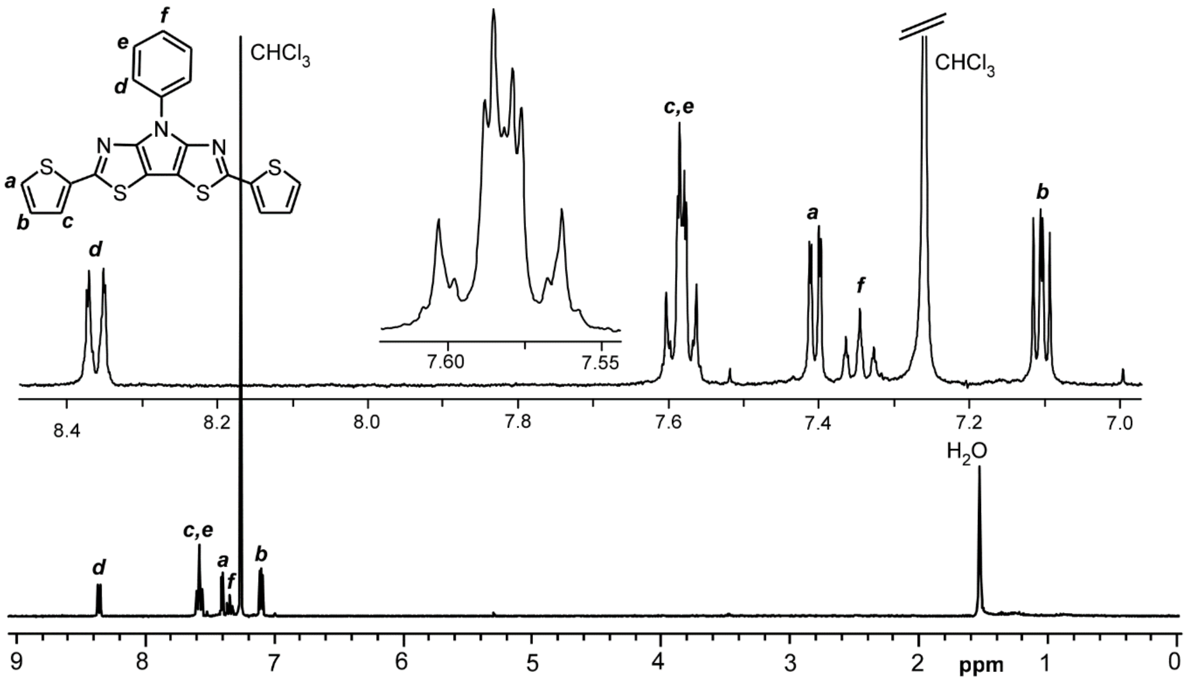

Figure S7. ^{1}H NMR Spectrum of 4-phenyl-2,6-bis(2-thienyl)-4*H*-pyrrolo[2,3-*d*:5,4-*d'*]bisthiazole.

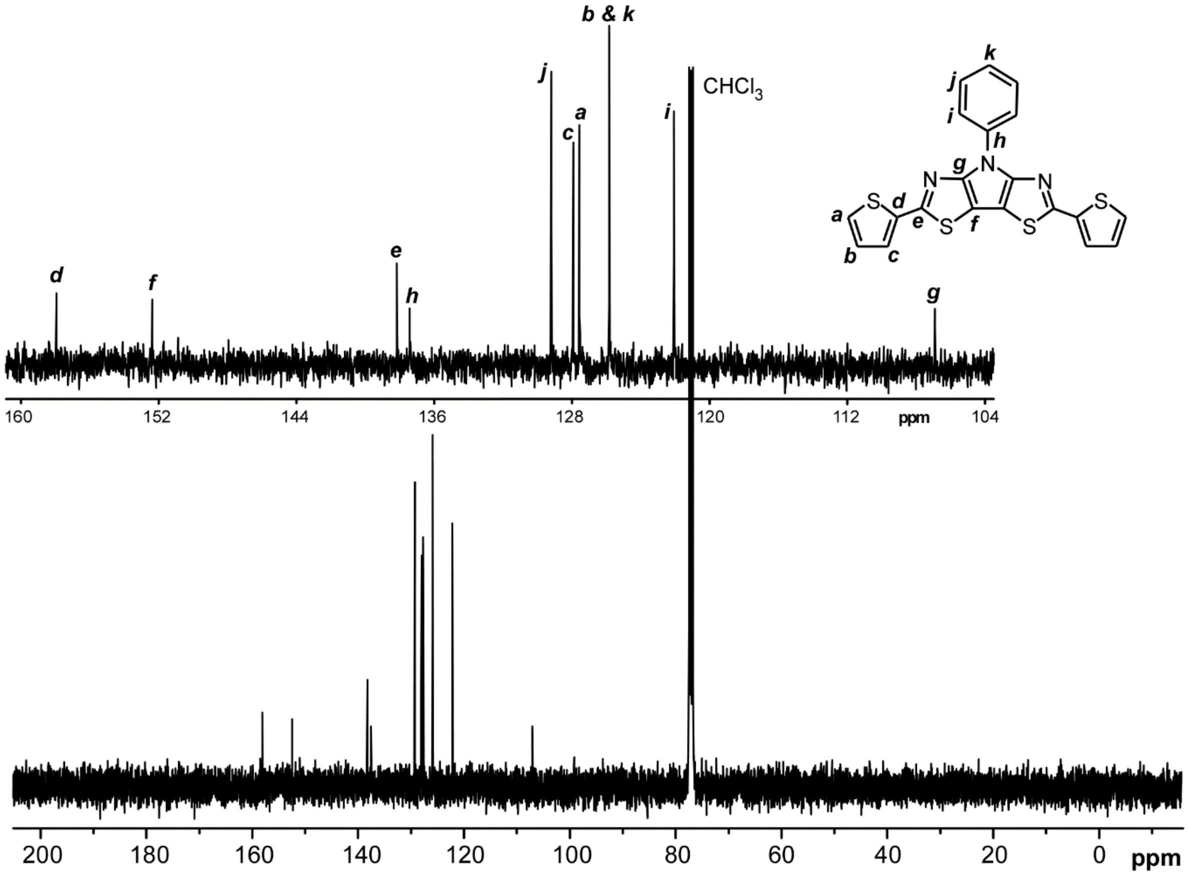

Figure S8. ^{13}C NMR Spectrum of 4-phenyl-2,6-bis(2-thienyl)-4*H*-pyrrolo[2,3-*d*:5,4-*d'*]bisthiazole.

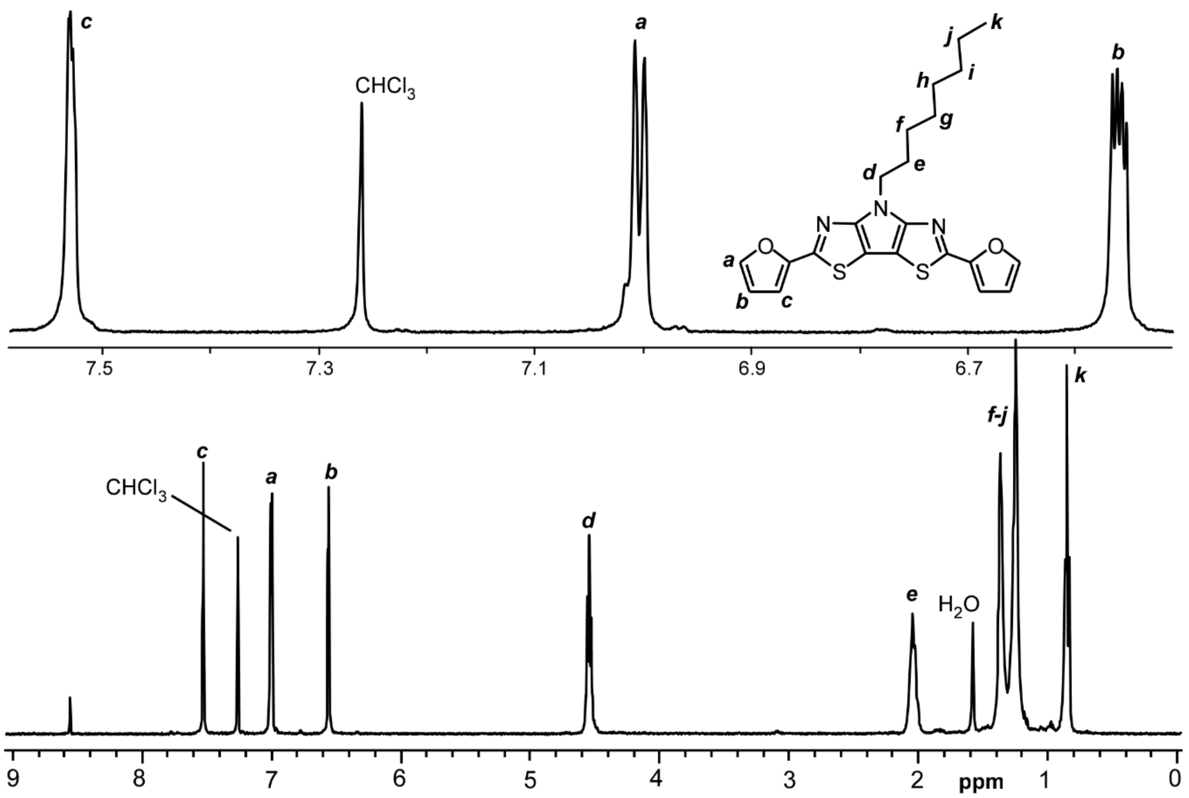

Figure S9. ^{1}H NMR Spectrum of 2,6-bis(2-furyl)-4-octyl-4*H*-pyrrolo[2,3-*d*:5,4-*d'*]bisthiazole.

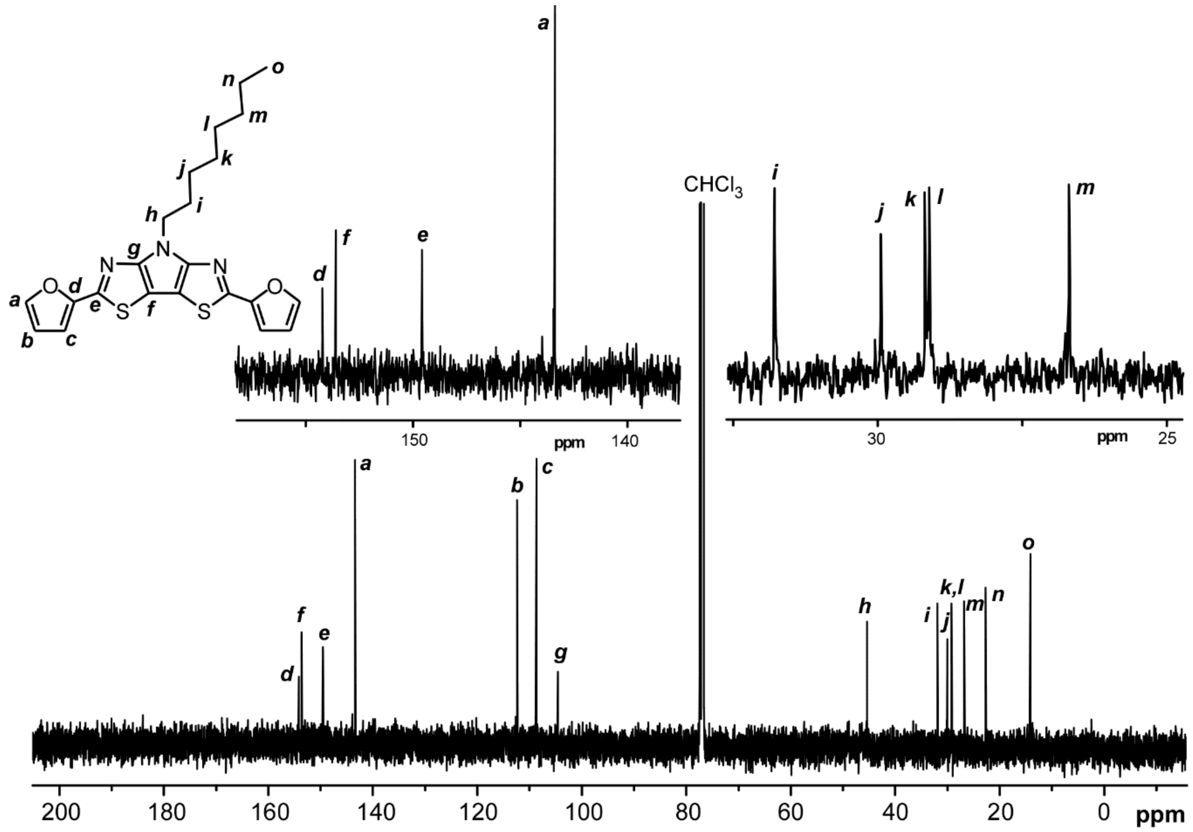

Figure S10. ^{13}C NMR Spectrum of 2,6-bis(2-furyl)-4-octyl-4*H*-pyrrolo[2,3-*d*:5,4-*d'*]bisthiazole.

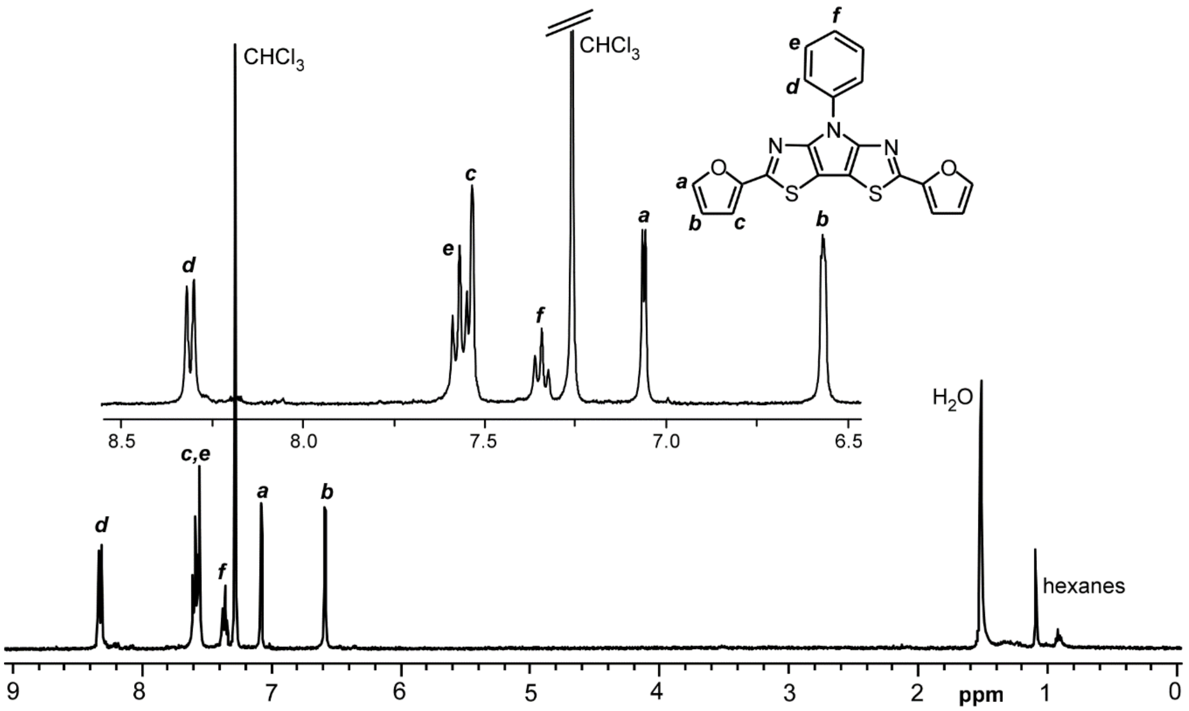

Figure S11. ^{1}H NMR Spectrum of 2,6-bis(2-furyl)-4-phenyl-4*H*-pyrrolo[2,3-*d*:5,4-*d′*]bisthiazole.

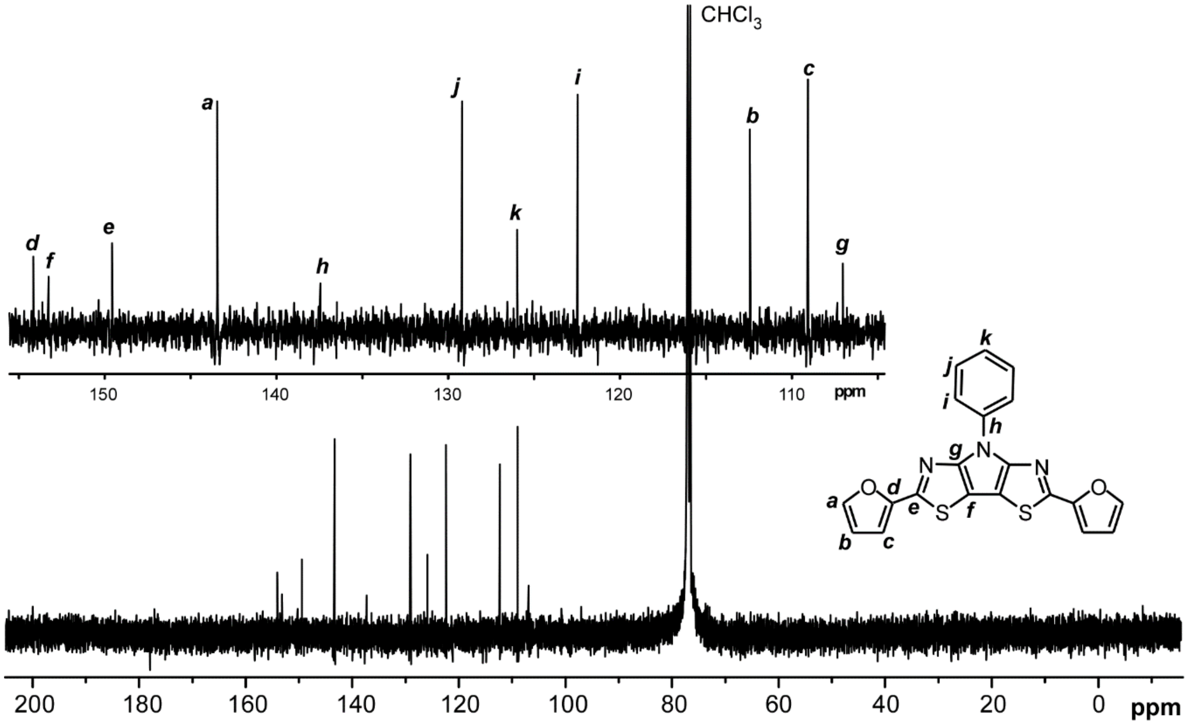

Figure S12. ^{13}C NMR Spectrum of 2,6-bis(2-furyl)-4-phenyl-4*H*-pyrrolo[2,3-*d*:5,4-*d′*]bisthiazole.

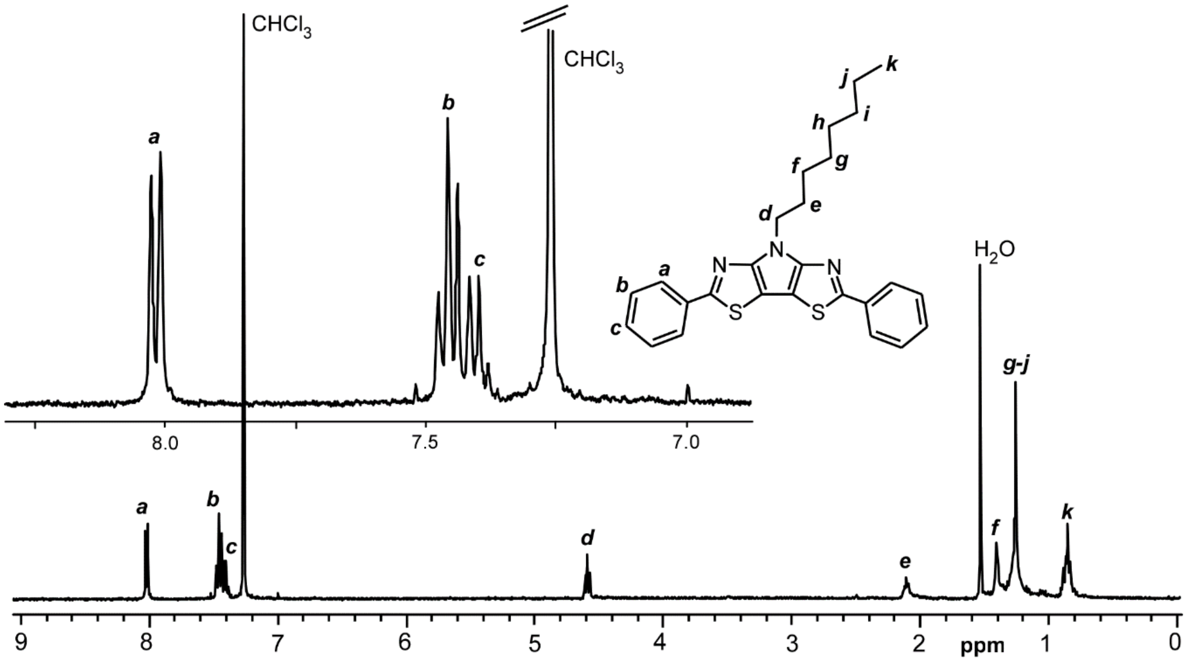

Figure S13. ^{1}H NMR Spectrum of 4-octyl-2,6-diphenyl-4*H*-pyrrolo[2,3-*d*:5,4-*d′*]bisthiazole.

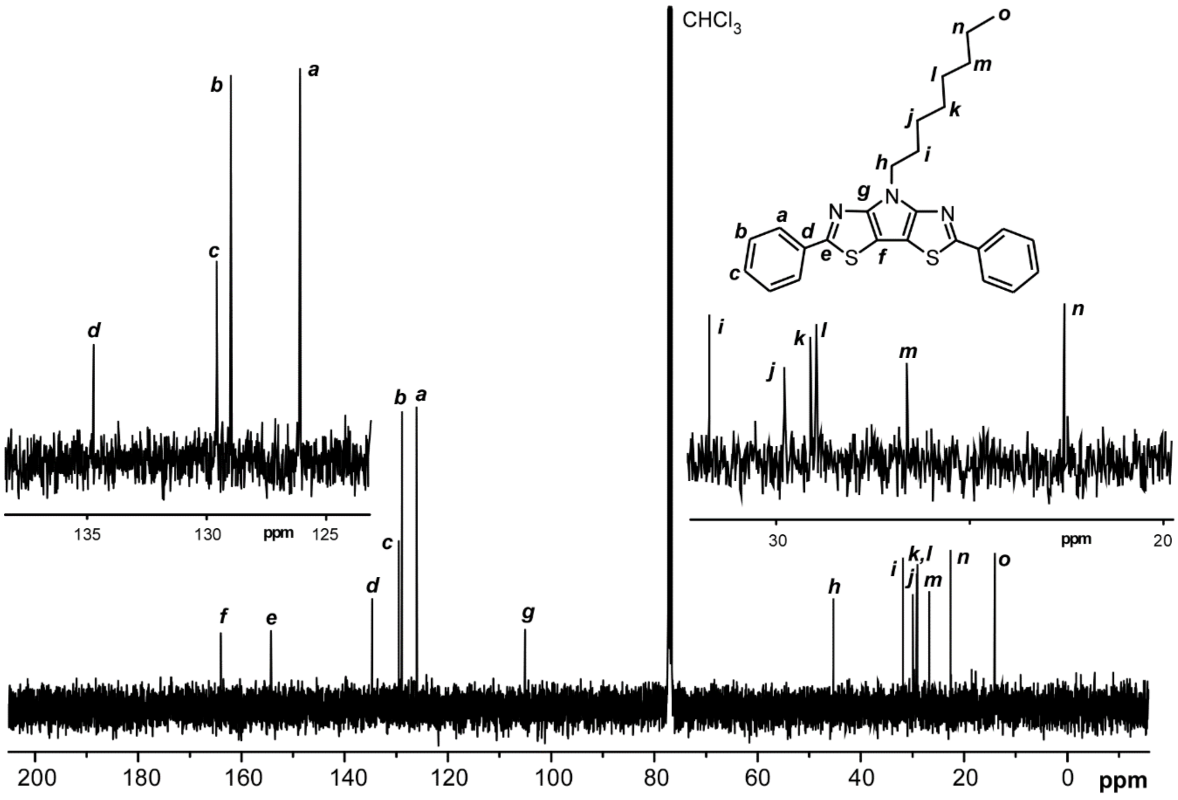

Figure S14. ^{13}C NMR Spectrum of 4-octyl-2,6-diphenyl-4*H*-pyrrolo[2,3-*d*:5,4-*d′*]bisthiazole.

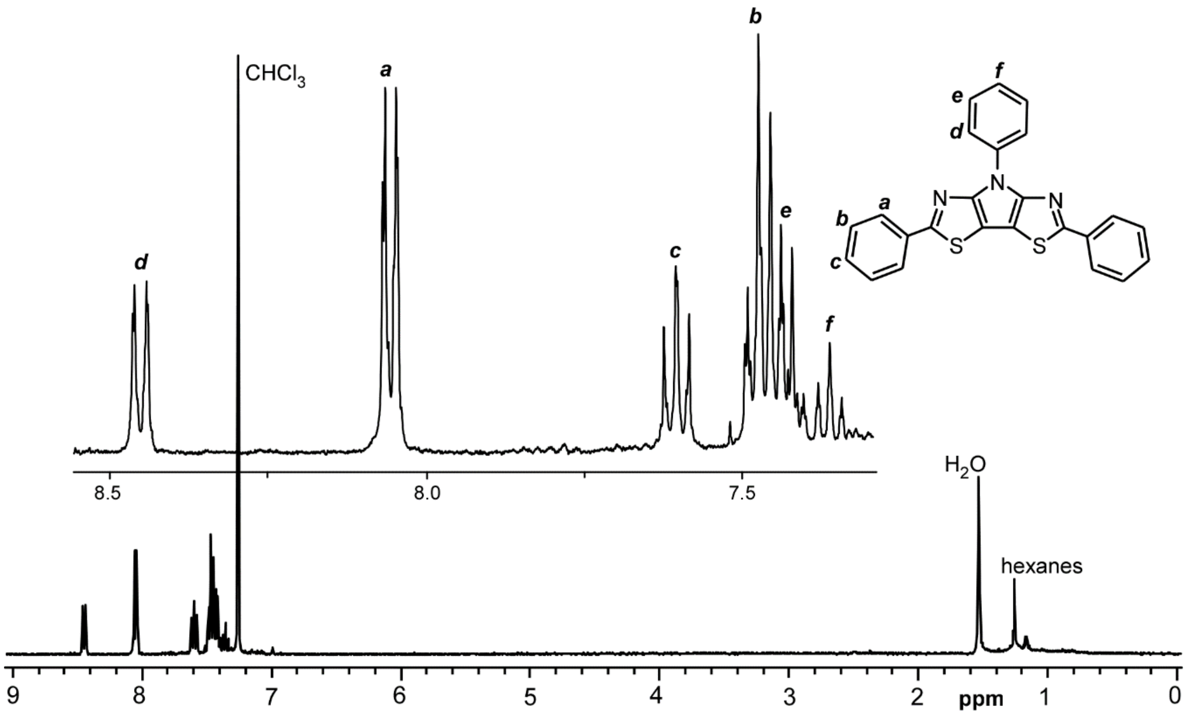

Figure S15. ^{1}H NMR Spectrum of 2,4,6-triphenyl-4*H*-pyrrolo[2,3-*d*:5,4-*d′*]bisthiazole.

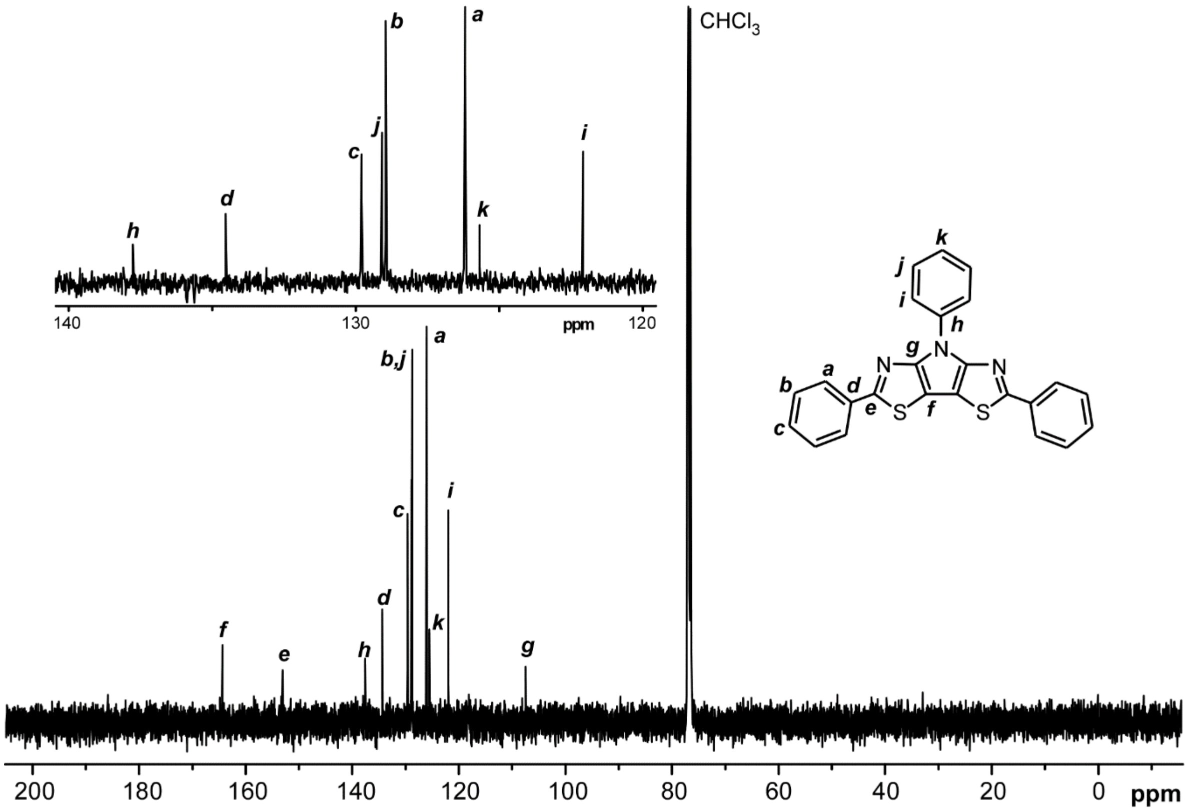

Figure S16. ^{13}C NMR Spectrum 2,4,6-triphenyl-4*H*-pyrrolo[2,3-*d*:5,4-*d′*]bisthiazole.

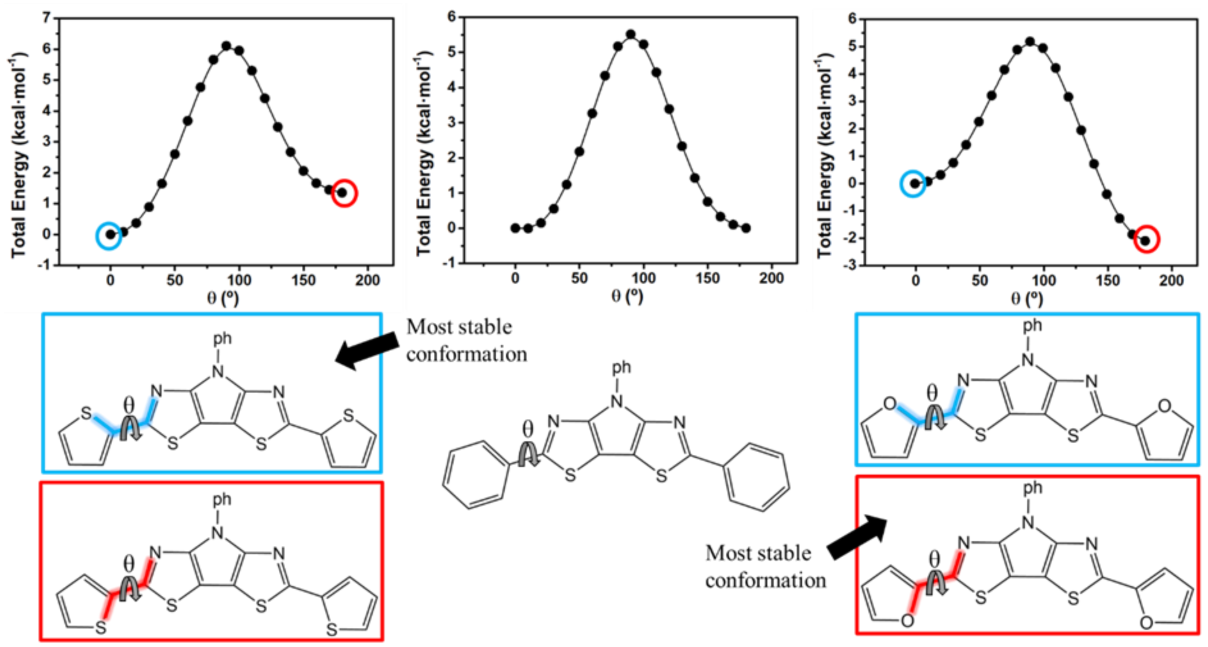

Figure S17. Rotational analysis calculated at the B3LYP/6-31G** level.

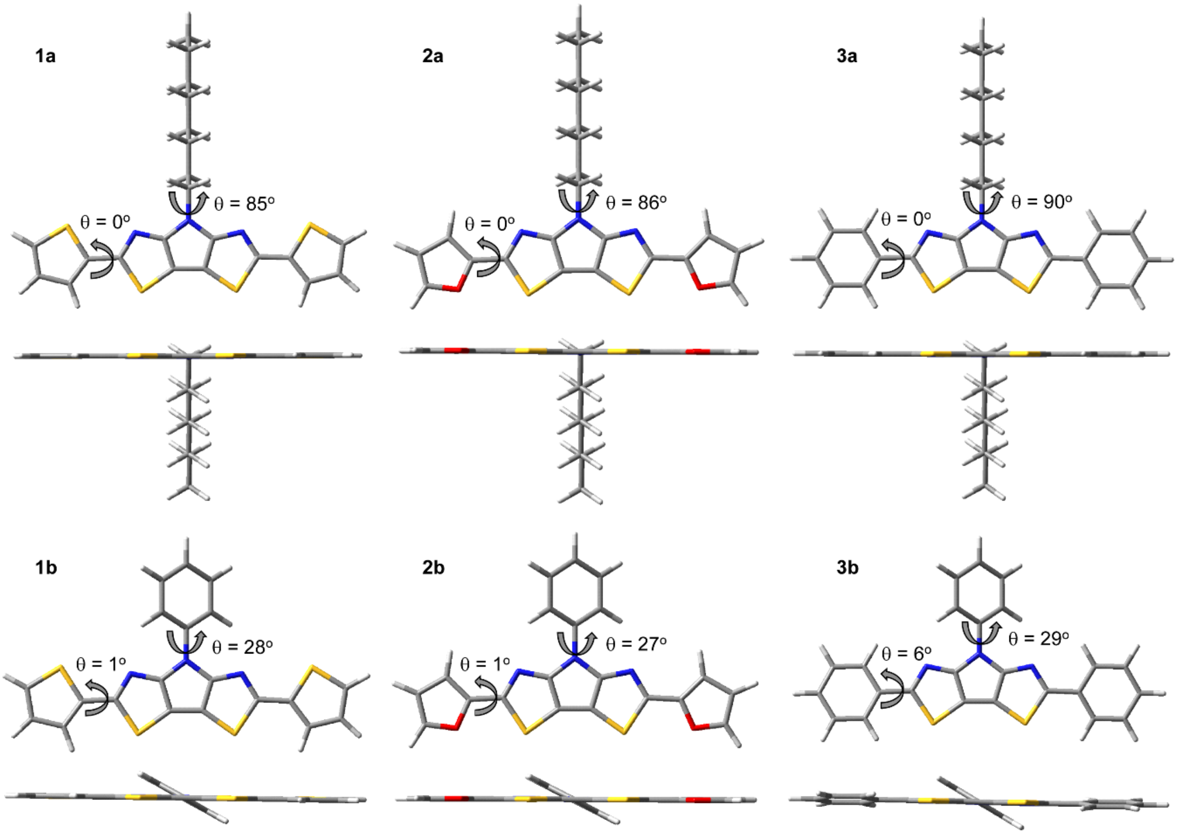

Figure S18. Optimized structures calculated at the B3LYP/6-31G** level.

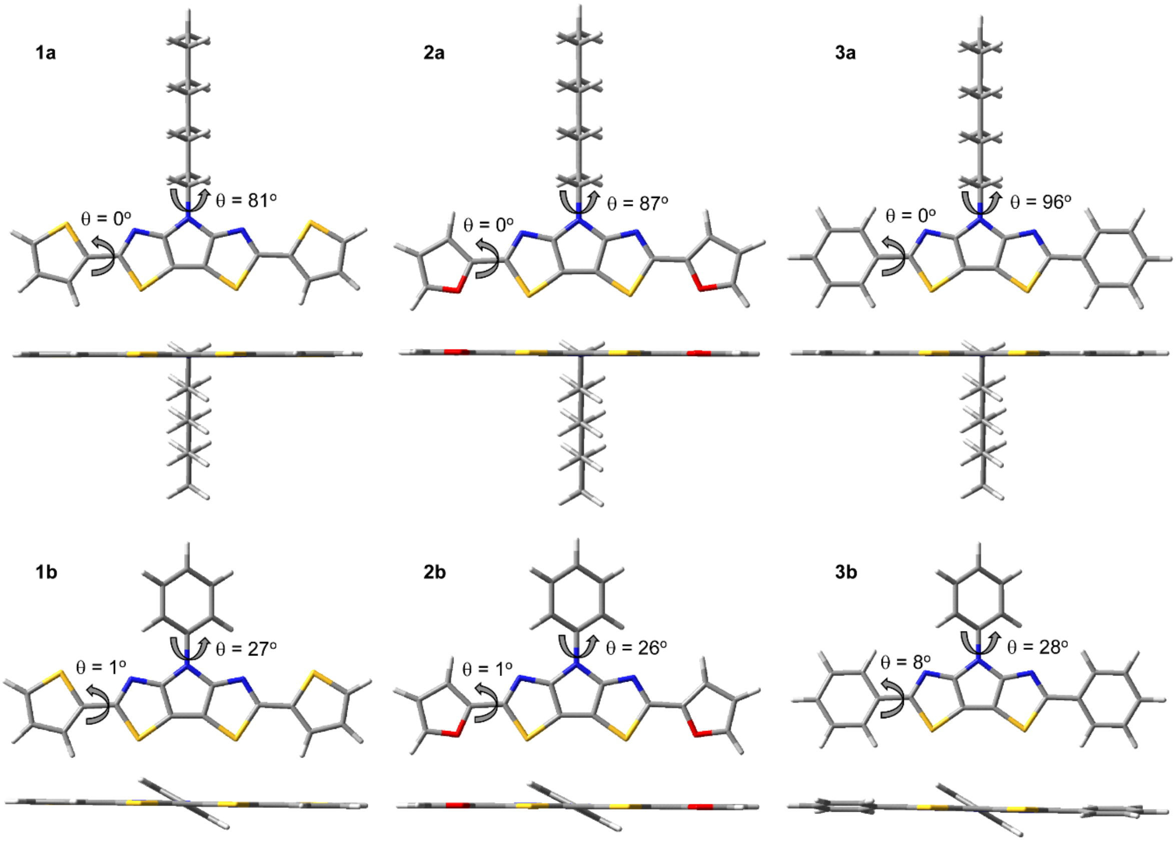

Figure S19. Optimized structures calculated at the CAM-B3LYP/6-31G** level.

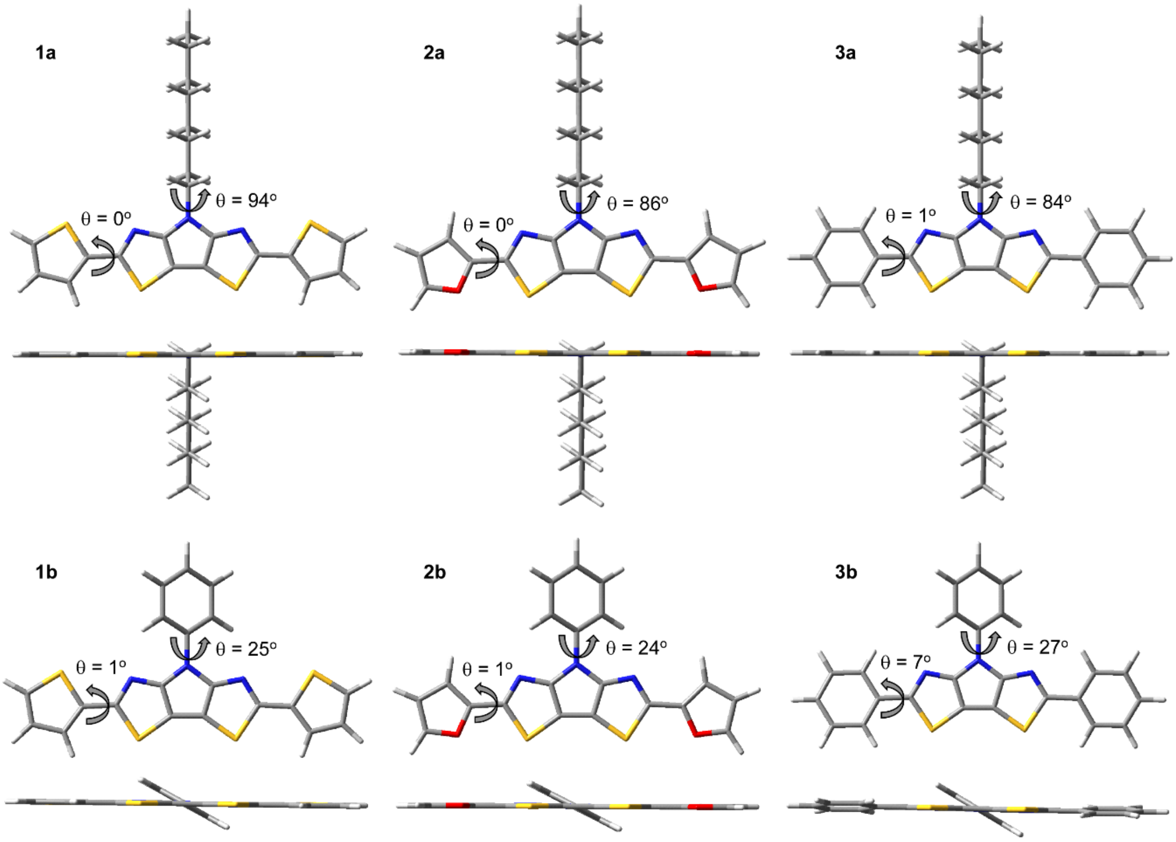

Figure S20. Optimized structures calculated at the PBE0/6-31G** level.

Table S1. DFT-calculated frontier orbital energies and vertical ionization potentials at the CAM-B3LYP/6-31G** level.

Oligomer	HOMO (eV)	LUMO (eV)	Vertical IP (eV)
**1a**	-6.24	-0.80	6.53 (5.86)
**1b**	-6.39	-0.92	6.65 (5.99)
**2a**	-6.14	-0.56	6.46 (5.63)
**2b**	-6.30	-0.71	6.59 (5.84)
**3a**	-6.33	-0.63	7.53 (6.76)
**3b**	-6.50	-0.75	7.37 (6.63)

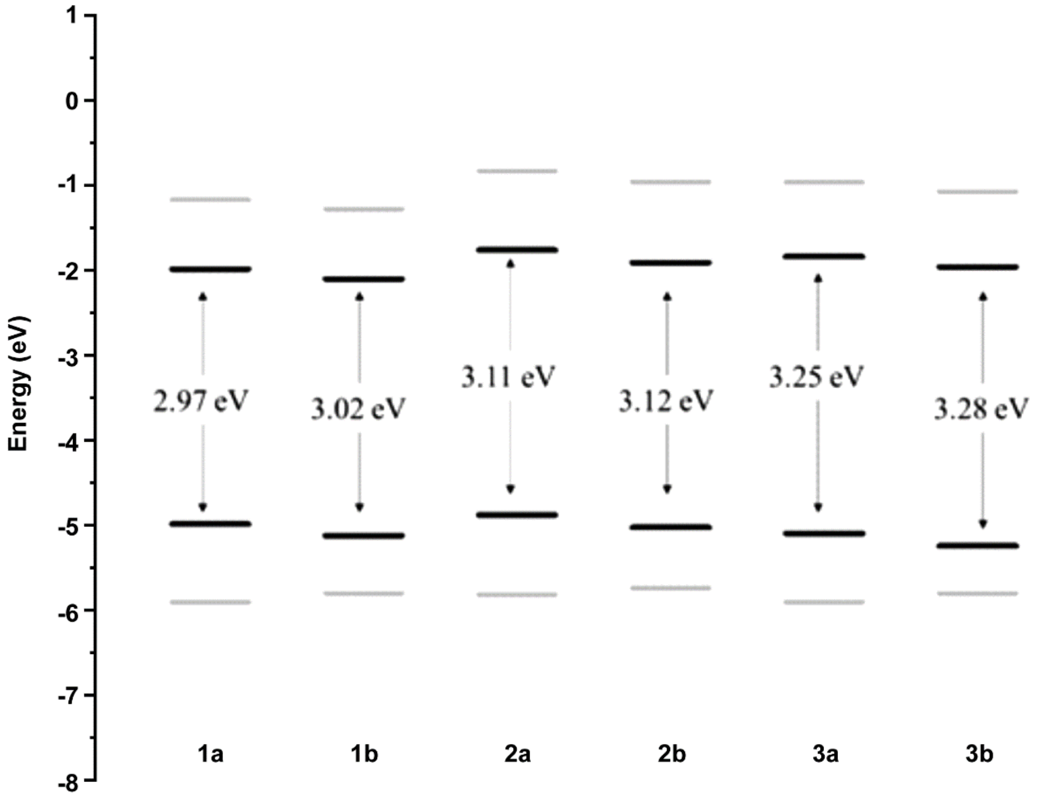

Figure S21. Frontier orbital energies calculated at the B3LYP/6-31G** level. The HOMO-LUMO gap values are also shown.

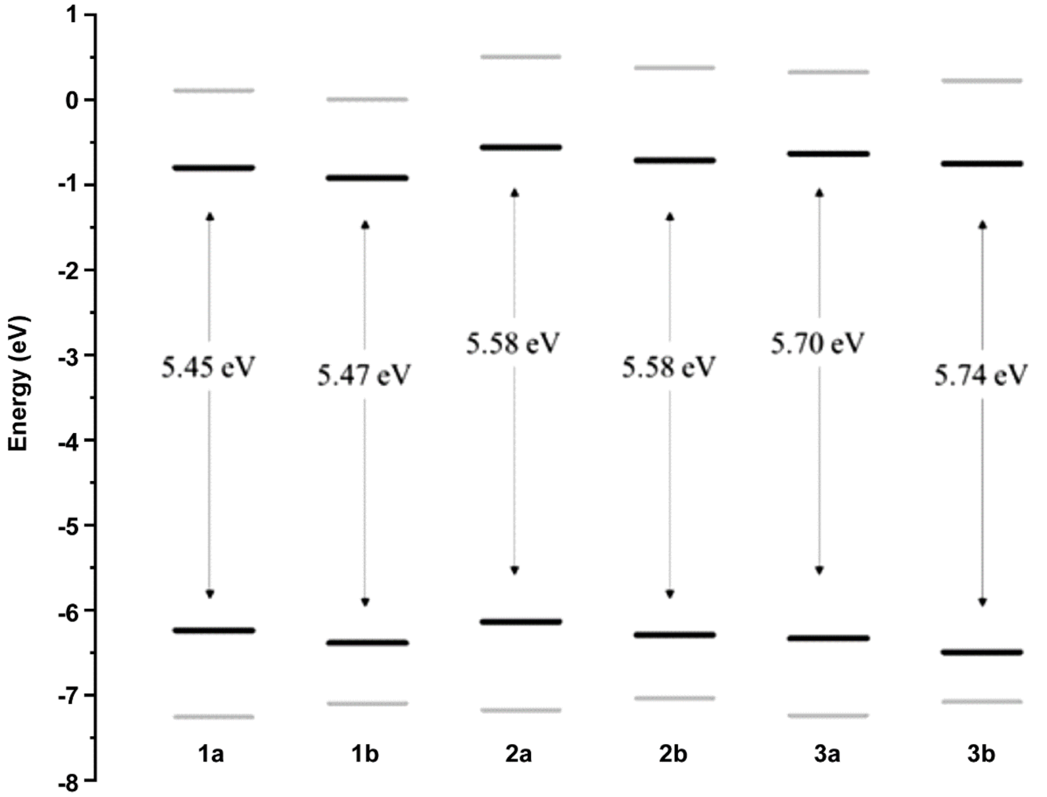

Figure S22. Frontier orbital energies calculated at the CAM-B3LYP/6-31G** level. The HOMO-LUMO gap values are also shown.

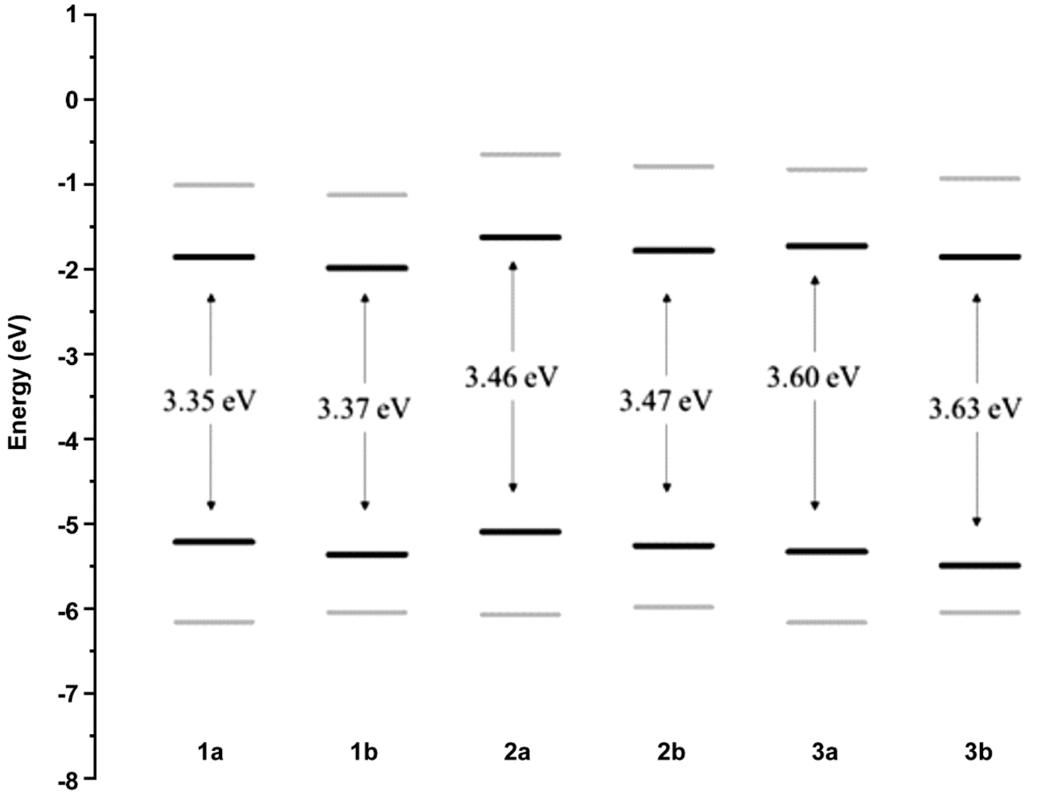

Figure S23. Frontier orbital energies calculated at the PBE0/6-31G** level. The HOMO-LUMO gap values are also shown.

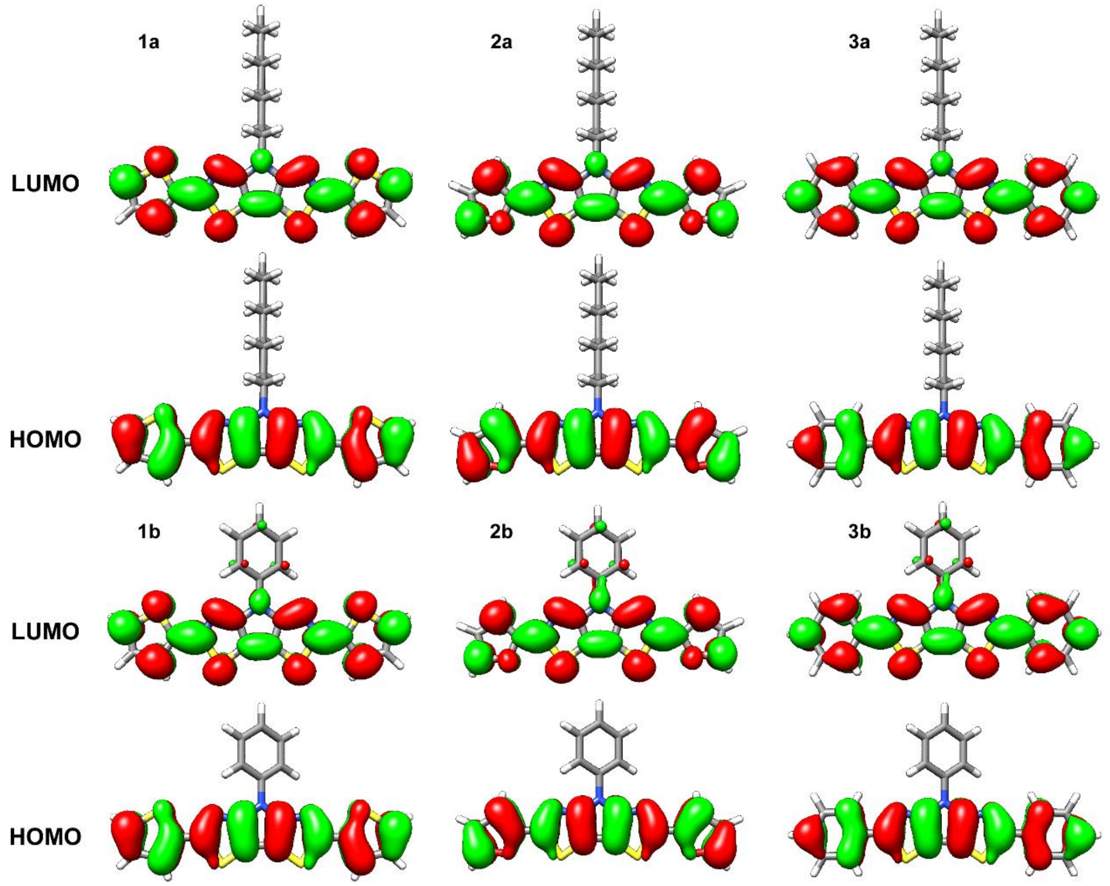

Figure S24. Molecular orbital topologies calculated at the B3LYP/6-31G** level.

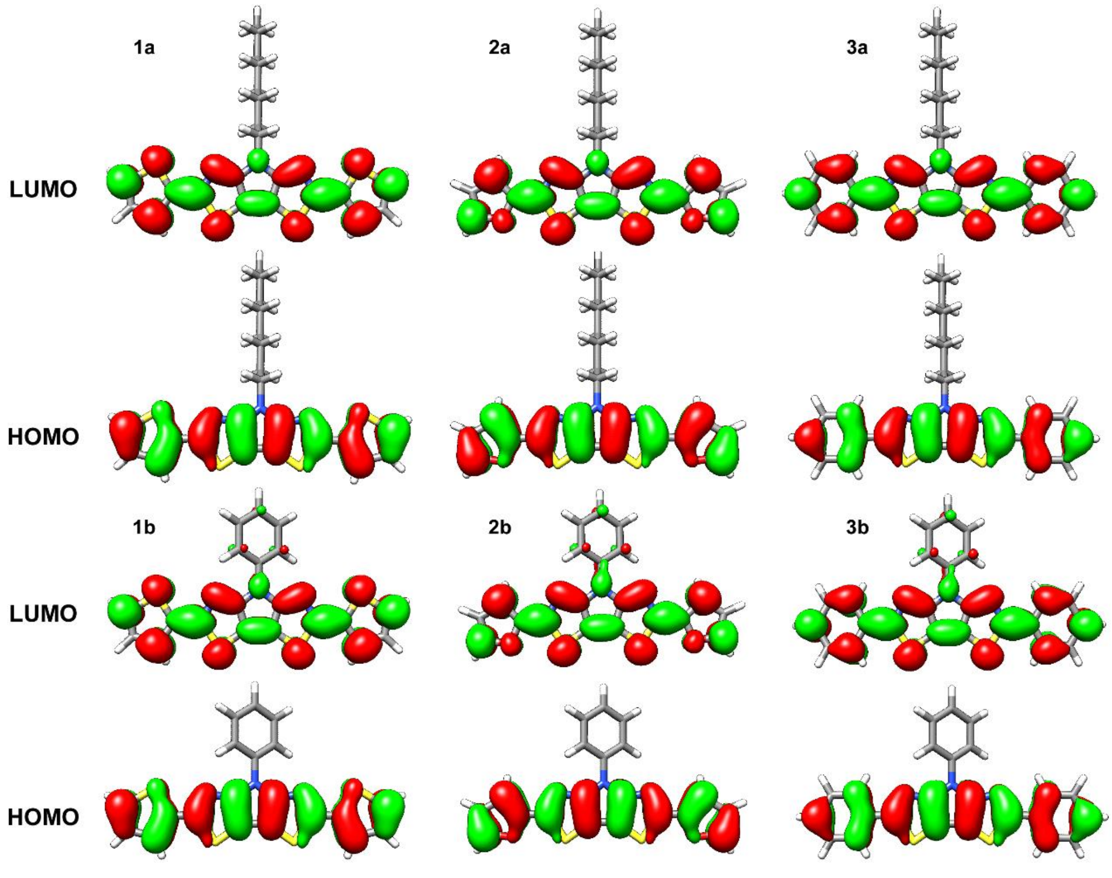

Figure S25. Molecular orbital topologies calculated at the CAM-B3LYP/6-31G** level.

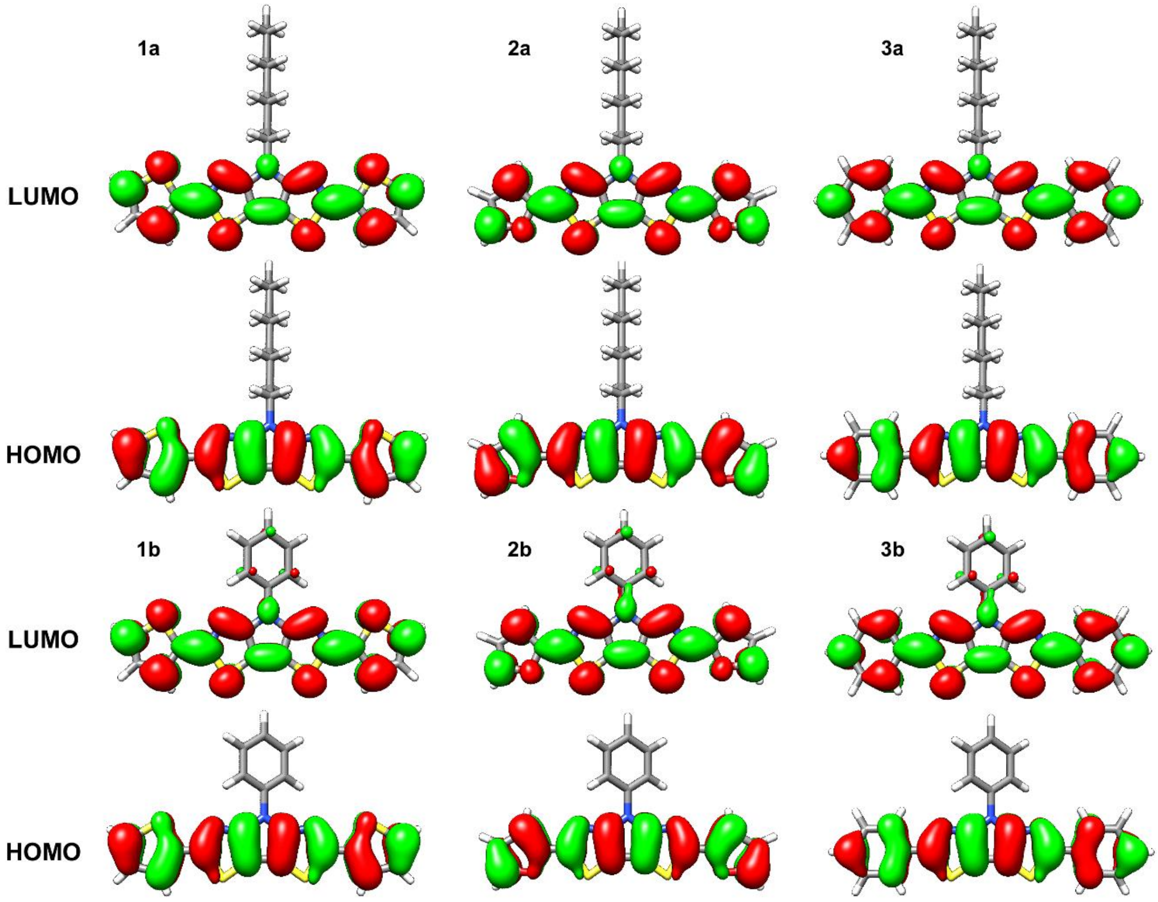

Figure S26. Molecular orbital topologies calculated at the PBE0/6-31G** level.

Table S2. DFT-calculated vertical excitation energies at the B3LYP/6-31G** and CAM-B3LYP/6-31G** levels.

Oligomer	B3LYP/6-31G**			CAM-B3LYP/6-31G**		
	E_{max} (eV)	f	Description	E_{max} (eV)	f	Description
**1a**	2.85	1.26	HOMO→LUMO (100%)	3.32	1.25	HOMO→LUMO (94%)
**1b**	2.86	1.21	HOMO→LUMO (100%)	3.33	1.22	HOMO→LUMO (94%)
**2a**	3.00	1.30	HOMO→LUMO (100%)	3.44	1.30	HOMO→LUMO (95%)
**2b**	2.99	1.24	HOMO→LUMO (100%)	3.44	1.26	HOMO→LUMO (95%)
**3a**	3.05	1.31	HOMO→LUMO (100%)	3.52	1.30	HOMO→LUMO (95%)
**3b**	3.07	1.25	HOMO→LUMO (100%)	3.54	1.25	HOMO→LUMO (95%)

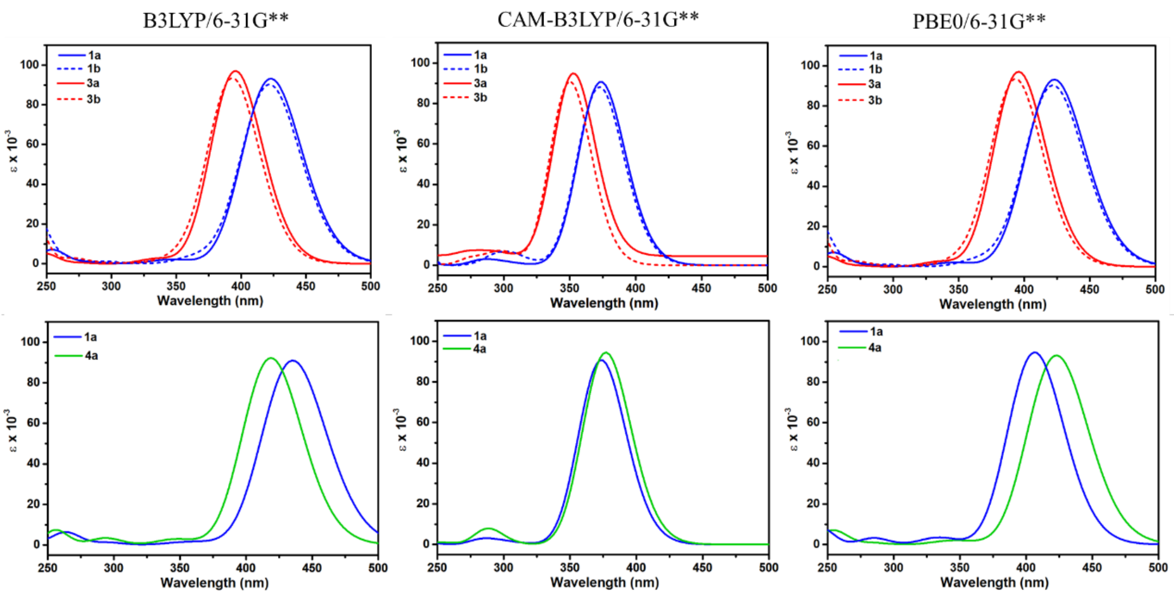

Figure S27. Simulated UV-visible spectra at B3LYP/6-31G**, CAM-B3LYP/6-31G** and PBE0/6-31G** levels.

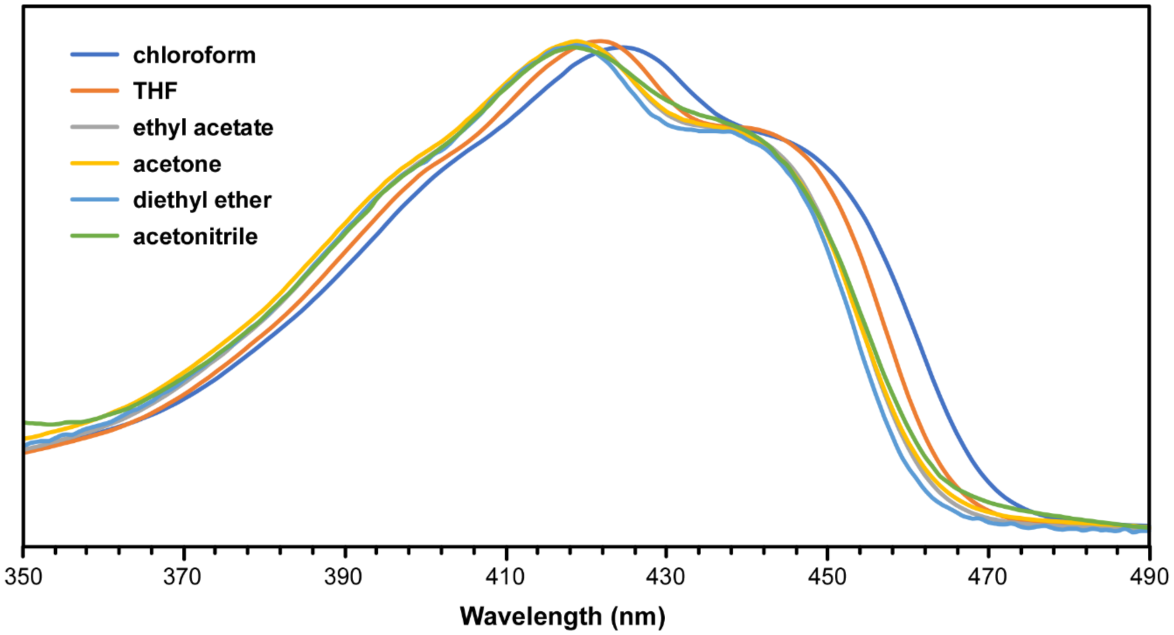

Figure S28. Solution UV-visible spectra of **1a** in various solvents.
